# Computational Prediction of RNA–RNA Interactions between Small RNA Tracks from *Betacoronavirus* Nonstructural Protein 3 and Neurotrophin Genes during Infection of an Epithelial Lung Cancer Cell Line: Potential Role of Novel Small Regulatory RNA

**DOI:** 10.3390/v15081647

**Published:** 2023-07-28

**Authors:** Alexis Felipe Rojas-Cruz, Clara Isabel Bermúdez-Santana

**Affiliations:** 1 Theoretical and Computational RNomics Group, Department of Biology, Faculty of Sciences, Universidad Nacional de Colombia, Bogotá 111321, Colombia; alrojascr@unal.edu.co; 2 Center of Excellence in Scientific Computing, Universidad Nacional de Colombia, Bogotá 111321, Colombia

**Keywords:** *Betacoronavirus*, RNA–RNA interaction, virus-derived small RNAs, host miRNA machinery, human lung physiopathology, neurotrophin signaling impairment

## Abstract

Whether RNA–RNA interactions of cytoplasmic RNA viruses, such as *Betacoronavirus*, might end in the biogenesis of putative virus-derived small RNAs as miRNA-like molecules has been controversial. Even more, whether RNA–RNA interactions of wild animal viruses may act as virus-derived small RNAs is unknown. Here, we address these issues in four ways. First, we use conserved RNA structures undergoing negative selection in the genomes of SARS-CoV, MERS-CoV, and SARS-CoV-2 circulating in different bat species, intermediate animals, and human hosts. Second, a systematic literature review was conducted to identify *Betacoronavirus*-targeting hsa-miRNAs involved in lung cell infection. Third, we employed sophisticated long-range RNA–RNA interactions to refine the seed sequence homology of hsa-miRNAs with conserved RNA structures. Fourth, we used high-throughput RNA sequencing of a *Betacoronavirus*-infected epithelial lung cancer cell line (Calu-3) to validate the results. We proposed nine potential virus-derived small RNAs: two vsRNAs in SARS-CoV (Bats: SB-vsRNA-ORF1a-3p; SB-vsRNA-S-5p), one vsRNA in MERS-CoV (Bats: MB-vsRNA-ORF1b-3p), and six vsRNAs in SARS-CoV-2 (Bats: S2B-vsRNA-ORF1a-5p; intermediate animals: S2I-vsRNA-ORF1a-5p; and humans: S2H-vsRNA-ORF1a-5p, S2H-vsRNA-ORF1a-3p, S2H-vsRNA-ORF1b-3p, S2H-vsRNA-ORF3a-3p), mainly encoded by nonstructural protein 3. Notably, *Betacoronavirus*-derived small RNAs targeted 74 differentially expressed genes in infected human cells, of which 55 upregulate the molecular mechanisms underlying acute respiratory distress syndrome (ARDS), and the 19 downregulated genes might be implicated in neurotrophin signaling impairment. These results reveal a novel small RNA-based regulatory mechanism involved in neuropathogenesis that must be further studied to validate its therapeutic use.

## 1. Introduction

Over the last two decades, there have emerged highly pathogenic and deadly *Betacoronaviruses* (*Beta-CoVs*), including severe acute respiratory syndrome coronavirus (SARS-CoV) and Middle East respiratory syndrome coronavirus (MERS-CoV), which can cause severe human disease but have only been observed in a one-time event and limited outbreaks [1,2]. More recently, a novel *Beta-CoV* named SARS-CoV-2 has been identified as the source of coronavirus disease 2019 (COVID-19), which continues to threaten the lives of hundreds of millions of people [3]. These positive-sense, single-stranded RNA (+ ssRNA) viruses belong to the *Beta-CoV* genus and carry one of the largest RNA genomes (~30 kilobases, kb) capped at the 5′ ends and poly-A tail among all RNA virus families [4,5,6]. Upon cell entry, genomic RNA is initially translated to produce nonstructural proteins from ORF1a and ORF1b and also serves as a template for the generation of subgenomic RNAs (sgRNAs) from the 3′ end, which are subsequently capped and translated into structural and accessory proteins [7,8]. Since the linear sequence of *Beta-CoV* genomes encodes all the proteins needed to take over the host cell machinery, the most interesting hallmark of these viruses is to understand how RNA molecules fold into complex, higher-conserved structures with potential RNA–RNA interactions that may perhaps give rise to virus-derived small RNAs (vsRNAs) with functions to modulate the host transcriptome that remain unknown. For this purpose, it is crucial to consider that RNA structures are typically subject to selection pressures. A well-conserved RNA structure indicates that, despite suffering an excess of noncompensatory changes during the cross-species barriers of *Beta-CoVs* (e.g., GC → CG or AU → UA), RNA structure-preserving supports maintained pointing to negative selection, whereas substitutions that alter base pairs (e.g., GC → AU or CG → UA) hint at relaxed constraints or positive selection [9,10,11].

During the last two decades, small noncoding RNAs (20–22 nt), including microRNAs (miRNAs), have attracted attention owing to their well-conserved structure and regulatory role in gene expression [12,13]. In a eukaryotic context, the biogenesis of human miRNAs (hsa-miRNAs) is transcribed in the nucleus from RNA polymerase II into long primary transcripts (pri-miRNAs). The nuclear microprocessor machinery (Drosha/DGCR8) cleaves pri-miRNAs into small precursor-miRNAs (pre-miRNAs) that are then exported into the cytoplasm by an exportin 5 (XPO5)/RanGTP complex. In the cytoplasm, they are further processed to become mature miRNAs by Dicer, an RNase III-type protein, and loaded into the Argonaute (AGO) protein to produce the effector RNA-induced silencing complex (RISC) [14]. The RNA–RNA binding of a microRNA and its target typically involves perfect complementarity between a sequence of six to eight bases at the 5′ end of the mature miRNA, known as the miRNA seed, and a cognate complementary sequence in the target’s 3′ untranslated region [15,16]. In light of these considerations, viruses often hijack the miRNA pathway by clearing host miRNAs or producing their miRNAs as viral microRNAs (v-miRNAs) [17,18]. Since some enzymes are involved in the biogenesis of hsa-miRNAs located in the nucleus [19], all known v-miRNAs are derived from RNA viruses that replicate in the nucleus [20]. Recent reports have identified v-miRNAs in HIV-1 (miR-N367, vmiR88, vmiR89 y miR-H3-3p) [21,22,23], influenza (miR-HA-3p) [24], dengue-2 (DENV–vsRNA-5) [25], and Ebola (miR-VP-3p, Zebov-miR-1-5p y miR-T1-5p) [26,27,28,29]. Regarding cytoplasmic RNA viruses, whether or not these viruses produce v-miRNAs is not yet fully understood. However, previous studies have suggested that bearing artificial miRNA sequences may be a source of v-miRNAs [30,31,32].

*Beta-CoVs* replicate into the cytoplasm, and most studies rely on the assumption that v-miRNAs derived from their genomes are processed by the host miRNA pathway [33,34,35,36,37]. Whether *Beta-CoVs* produce functional v-miRNAs has been controversial for several years [38]. Here, conserved RNA structures with strong negative selection signals (*s* ≤ 2.99), recently identified in a wide range of *Beta-CoVs* circulating in bats, a variety of animal species, and humans [39], are proposed as model RNA structures to unravel potential RNA–RNA interactions with the capacity to function as vsRNAs in host gene regulation. Additionally, putative vsRNAs are validated using bulk RNA sequencing (RNA-seq) of the epithelial lung cancer cell line Calu-3 infected with SARS-CoV, MERS-CoV, and SARS-CoV-2. The RNA–RNA binding for predicting vsRNAs as functional miRNA-like molecules is challenging. Still, our approach promises to be highly reliable and novel, shedding light on the mechanisms and possible role of vsRNAs in the pathogenesis for developing antago-miR therapies against these pathogenic viruses.

## 2. Materials and Methods

### 2.1. Data Acquisition

We retrieved conserved RNA structures under negative selection (*s* ≤ 2.99) in *Beta-CoV* genomes that were found in bats, intermediate animals, and humans from a recent study [39]. Briefly, the prediction of structured regions across the whole viral genome was scanned in windows of length 120 nucleotides sliding by 40 nucleotides with RNAz (v2.1) [40]. Conserved RNA structures with *p* > 0.98, and z < −3 were retrieved and then tested to estimate their selection pressures using SSS-test (v1.0) [11].

RNA-seq expression profiling was obtained from the Gene Expression Omnibus (GEO) database (NCBI Reference series: GSE148729) of the epithelial lung cancer cell line (Calu-3) in mock and infected with SARS-CoV (GER-FRA/2003) and SARS-CoV-2 (USA-WA1/2020). For MERS-CoV, a complete transcriptional profile (RNA-seq) of circRNA, miRNA, and mRNA expression was analyzed in epithelial lung cancer cells (Calu-3) for mock and infected with MERS-CoV (HCoV-EMC/2012), downloaded from the GEO database (NCBI Reference series: GSE139516).

### 2.2. Differential Expression Analysis

Gene-level differential expression analysis was carried out using the edgeR package (v3.36) in R [41] to determine the set of differentially expressed genes (DEGs) as a response to the viral infection. All samples were prefiltered to discard genes with a read count less than 2, and then for each group, genes with a minimum requirement of 20 counts per million (CPM) across libraries were kept. Count data were then normalized using trimmed means of M values (TMM) to adjust for sequencing library size difference [42]. For each virus, three comparisons were performed: (i) mock cells between the final and initial time (MM), (ii) infected cells upon post-infection against onset (II), and (iii) a comparison of infected cells with the respective mock cells (IM). We employed a quasi-likelihood F test to assess the significance of group differences [42]. Significant DEGs were defined with |log_2_ (fold change, FC)| > 1.5 and false discovery rate (FDR)-adjusted *p*-value < 0.05 using Benjamini–Hochberg’s procedure for multiple comparison adjustment.

### 2.3. hsa-miRNAs Targeting Beta-CoVs in Respiratory Epithelial Cells

Given that the upper respiratory tract probably represents the onset site for *Beta-CoV* infection [43], a systematic literature review was conducted to identify hsa-miRNAs involved in lung cell infection for SARS-CoV, MERS-CoV, and SARS-CoV-2. In this systematic review, databases such as Pubmed, Lilacs, EMBASE, Scopus, Web of Science Core Collection (WosCC), and EBSCO were searched using the terms “microRNA” and its synonyms (microRNA OR hsa-miRNA OR miR OR miRNA OR small noncoding RNA OR small ncRNA), “lung epithelial infection” (lung epithelial infection OR pulmonary epithelial infection OR respiratory epithelial infection) and in titles/abstracts as “SARS-CoV, MERS-CoV, and SARS-CoV-2”, separately. Research articles with either robust computational or experimental validation were considered for the inclusion of hsa-miRNAs until July 2022.

### 2.4. RNA–RNA Interactions

For each virus, the putative 3′UTR regions of genes targeted by hsa-miRNAs were predicted using TargetScan [44], miRDB [45], and miRTarBase [46], implemented in the miRWalk 3.0 [47] online tool with a binding probability ≥ 1.0. These binding pairs were cross-validated with intercepted DEGs between comparisons II and IM. For a rigorous target conservation analysis, hsa-miRNAs sequences were retrieved from miRBase [48], while 3′UTRs sequences of their respective targets as DEGs from the UTRdb database [49]. Thermodynamic interaction was undertaken for each hsa-miRNAs:3′UTR pairing, preventing certain hsa-miRNAs from binding to the 3′UTR of other DEGs. We conducted these hybridization duplexes using RNAhybrid [50], miRanda [51], and mirTarP [52]. RNAhybrid queries were considered with a strict binding to seed region (nucleotides 2–8), a minimum free energy (MFE) ΔG cutoff ≥ −18 kcal/mol, and a maximum bulge and internal loop length of 1. The parameters for miRanda included a hybridization alignment score ≥140 and an MFE ΔG cutoff ≤ −18 kcal/mol, and for mirTarP, a consecutive base match of 7 and an MFE ΔG cutoff ≤ −18 kcal/mol. Finally, we focused only on hsa-miRNAs overlapped by the three methods with the ggVennDiagram package (v2.1) [53] implemented in R.

### 2.5. Prediction of Putative vsRNAs

Upon the determination of potential hsa-miRNA candidates, we sought the most energetically favorable hybridization sites targeting conserved RNA structures with the negative selection at each host to identify putative vsRNAs derived from genomes of SARS-CoV, MERS-CoV, and SARS-CoV-2. The interactions between hsa-miRNA:viralRNA were predicted using RNAhybrid, miRanda, and mirTarP with similar parameters as specified in the hsa-miRNAs:3′UTR pairing. The hsa-miRNA binding site to viralRNA was considered as a possible candidate vsRNA.

### 2.6. Validation of vsRNAs and Prediction of Their Potential Targets

To verify whether there is a significant similarity between predicted vsRNAs and host miRNAs, a search of miRNAs deposited in miRbase 22 [48] based on the miRNA seed sequence was performed. Then, to better understand what processes may affect this subset of *Beta-CoV* vsRNAs, assuming that they are acting as host-gene regulators, human genes potentially targeted were identified using two independent tools including Diana (software MR-microT) [54], and miRDB (Custom prediction) [45]. Only predicted targets with a score ≥ 70 were considered in both cases. In addition, a reliable set of targets was obtained by overlapping predicted genes with the two algorithms. Finally, this list of targets was cross-validated with the repertoire of intercepted DEGs, and those overlapping were retrieved.

### 2.7. Functional Enrichment Analysis

The functionality of these DEGs was analyzed by an over-representation analysis (ORA) of gene ontology (GO) terms using Protein Analysis Through Evolutionary Relationships (PANTHER) [55]. GO terms enriched in biological process (BP) and Reactome pathways were considered using a Fisher’s exact tests with an FDR-adjusted *p*-value < 0.05. In addition, the Gene Cards database (https://www.genecards.org/, accessed on 16 March 2023) was used to reveal the human lung epithelial tissue, in which DEGs targeted by vsRNAs are expressed, and also to retrieve information on the pathologies that might be linked to these genes.

## 3. Results

### 3.1. Over 80% of Conserved RNA Structures in Genomes of SARS-CoV, MERS-CoV, and SARS-CoV-2 Are under Negative Selection

This study employs conserved RNA structures that showed negative selective pressures for genomes of the three *Beta-CoVs* (*s* ≤ 2.99) in a wide variety of hosts, including bats, intermediate animals, and humans. We retrieved 505 conserved loci or regions containing RNA structures acting under negative selection (Table 1). Among these loci, 133 (26%) were detected in SARS-CoV-2, followed by SARS-CoV and MERS-CoV with 159 (31%) and 213 (42%), respectively. We found that 81% of 619 loci under different selection pressures were conserved RNA structures with negative signals.

### 3.2. Identification of DEGs in Beta-CoV-Induced Epithelial Lung Cancer

**Figure 1:**
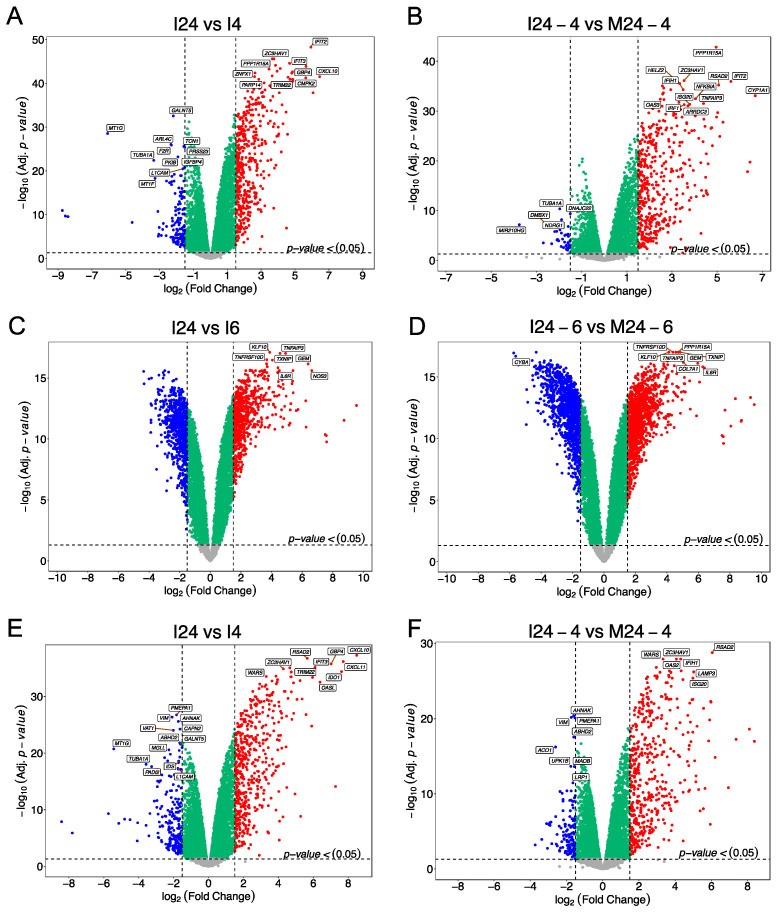
Volcano plots showing the differential expression profiling of Calu-3 cell cultures infected with *Beta-CoVs.* Differential analyses rely on the distribution of –log10 in the FDR-corrected *p*-value < 0.05 versus |log_2_FC | > 1.5 in (**A**,**B**) SARS-CoV, (**C**,**D**) MERS-CoV, and (**E**,**F**) SARS-CoV-2. For each virus, there are three comparisons: (i) mock cells between the final and initial time (MM) (Figure S2), (ii) infected cells upon post-infection against onset (II), and (iii) a comparison of infected cells with the respective mock cells (IM). Upregulated DEGs are shown as red dots and downregulated DEGs as blue dots.

To validate downstream analyses, we conducted a differential expression analysis using RNA-seq data obtained from Calu-3 cells infected with SARS-CoV-2, SARS-CoV, and MERS-CoV from the GEO database. Although in the first comparison (MM), SARS-CoV identified 172 DEGs (79 up- and 93 downregulated), MERS-CoV 115 DEGs (101 up- and 14 downregulated), and SARS-CoV-2 191 DEGs (86 up- and 105 downregulated) (Figure S2), the vast majority of DEGs resulted from the II and IM comparisons (Figure 1). For the II comparison, SARS-CoV registered 596 DEGs (461 up- and 135 downregulated), MERS-CoV 1622 DEGs (805 up- and 817 downregulated), and SARS-CoV-2 724 DEGs (505 up- and 219 downregulated), while in the IM comparison, 473 (431 up- and 42 downregulated), 2745 (1288 up- and 1457 downregulated), and 621 (480 up- and 141 downregulated) DEGs were detected in SARS-CoV, MERS-CoV, and SARS-CoV-2, respectively. 

**Figure 2:**
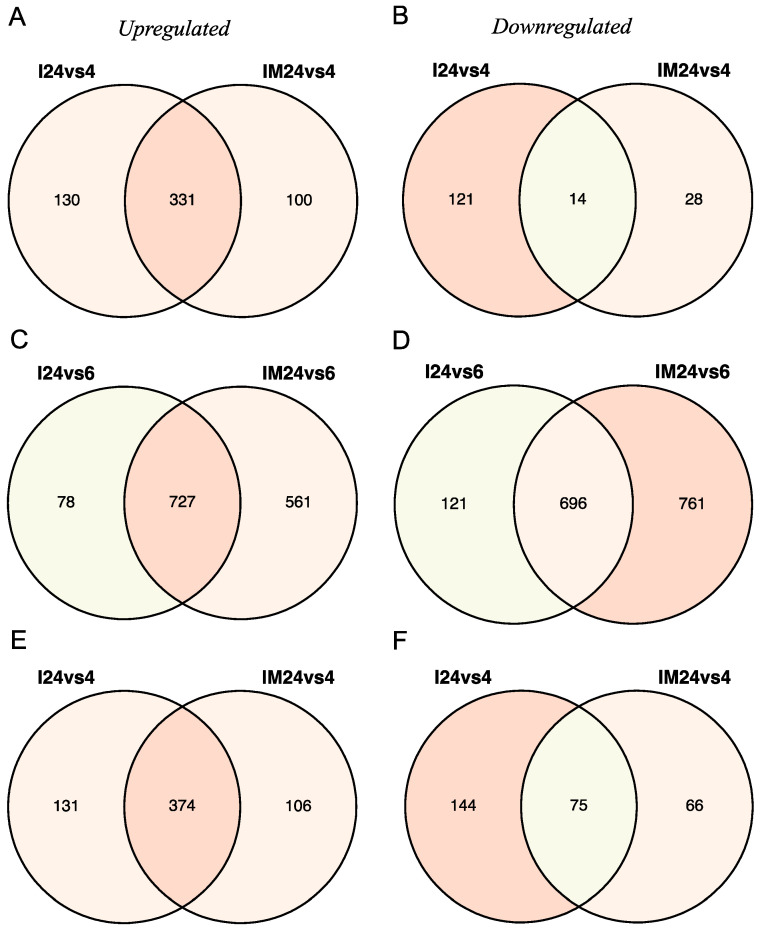
Venn diagrams register the amount of up- or downregulated DEGs in epithelial lung cancer cells infected with *Beta-CoVs*: (**A**,**B**) SARS-CoV; (**C**,**D**) MERS-CoV; and (**E**,**F**) SARS-CoV-2. For RNA–RNA interaction analyses, the intercepted DEGs between comparisons are considered: (i) mock cells between the final and initial time (MM) (no significant), (ii) infected cells upon post-infection against onset (II) and (iii) the comparison of infected cells with the respective mock cells (IM). Venn diagrams sections are colored based on the number of DEGs. The number of significant DEGs are shown for comparisons including 24 vs. 4 h post-infection and 24 vs. 6 h post-infection.

As the II and IM comparisons revealed the most significant number of DEGs, those in common were used for the validation of RNA–RNA interactions, target prediction, and functional enrichment. Accordingly, overlapping DEGs were identified, where 466 (331 up- and 135 downregulated) and 593 (374 up- and 219 downregulated) were accounted for SARS-CoV (Figure 2A,B), and SARS-CoV-2 (Figure 2E,F), respectively. Conversely, as MERS-CoV obtained the most considerable amount of DEGs, only 1423 (727 up- and 696 downregulated) were retrieved (Figure 2C,D)). The complete list of DEGs for the three viruses is available in Table S1.

### 3.3. hsa-miRNAs Associated with Lung Physiopathology in Beta-CoVs

For the prediction of putative RNA–RNA interactions, our strategy first consisted of searching research articles published until July 2022 that focused on describing the role, function, and/or association of hsa-miRNAs in the lung physiopathology of *Beta-CoVs.* Based on the literature review, we found a total of 256 hsa-miRNAs, including for SARS-CoV (36 hsa-miRNAs in 4 articles from 2002), MERS-CoV (70 hsa-miRNAs in 5 articles from 2012), and SARS-CoV-2 (150 hsa-miRNAs in 22 articles from 2019) (Table 2). Table S2 describes these hsa-miRNAs for each virus.

### 3.4. The let-7 Family of hsa-miRNAs Is the Most Frequently Predicted in hsa-miRNA:3′UTR Interactions

**Figure 3:**
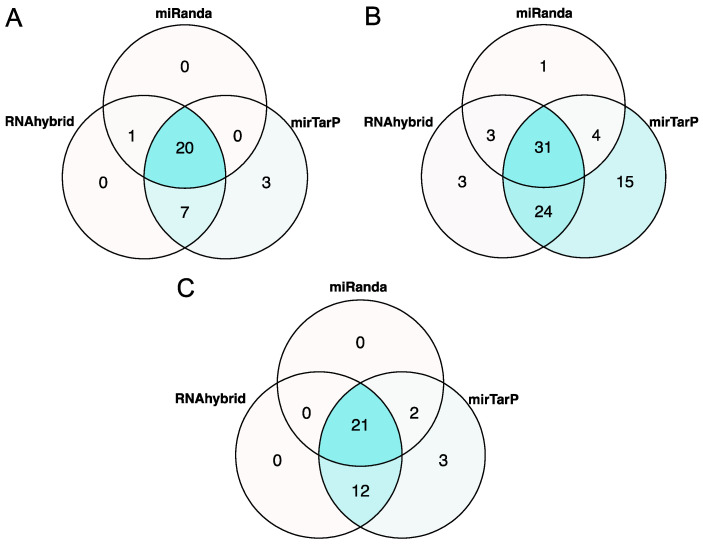
Potential common hsa-miRNA:3′UTR pairings predicted by RNAhybrid, miRanda, and mirTarP in *Beta-CoVs*: (**A**) There are 20 interaction pairs in SARS-CoV; (**B**) 31 in MERS-CoV; and (**C**) 21 in SARS-CoV-2. Venn diagram sections are colored based on the number of pairing predictions.

For each virus, hsa-miRNAs defined through the literature review were queried into miRWalk 3.0 to predict their putative 3′UTR regions of the target genes. Then, the hsa-miRNA:3′UTR pairings were cross-validated with previously identified DEGs, in which SARS-CoV obtained 52 pairings, MERS-CoV 210, and SARS-CoV-2 76, thus MERS-CoV had the most predicted pairings. To further decrease the impact of false favorable rates, the predicted interaction of hsa-miRNA:3′UTR was corroborated using scores from highly cited prediction tools such as RNAhybrid, miRanda, and mirTarP. These three tools matched 20 pairings in SARS-CoV, of which 15 (75%) showed to be of the let-7 family (Figure 3A). In a lower proportion, from 31 and 21 pairings of MERS-CoV and SARS-CoV-2, 19 (61%) and 5 (24%) concerned the same family of hsa-miRNAs, respectively (Figure 3B,C) (Table S3). In addition, it is worth mentioning that hsa-miRNA:3′UTR interactions showed a high binding homology to the seed sequence. For instance, for SARS-CoV, the predicted target sites were those where the MFE ranged from −19.4 to −30.7 kcal/mol, MERS-CoV between −18.0 and −30.6 kcal/mol, and SARS-CoV-2 between −18.2 and −32.2 kcal/mol (Table S3).

### 3.5. RNA–RNA Interactions in ORF1a Appear to Produce Discrete Putative vsRNAs

From the final hsa-miRNA:3′UTR interactions, hsa-miRNAs were retrieved to identify which binding sites are shared with the loci seed sequence showing negative selection in individual virus hosts using RNAhybrid, miRanda, and mirTarP (Figure S3). Interestingly, the analysis matched a repertoire of 25 hsa-miRNA:virus RNA pairings, where 20 of them were found in SARS-CoV-2, whereas MERS-CoV and SARS-CoV obtained 3 and 2 pairings, respectively (Table S4). However, there were hsa-miRNA:virus RNA interactions that matched identically in MERS-CoV and SARS-CoV-2, resulting in one and six pairings for each virus (Table S5). Finally, nine hybridization duplexes ranging from 18 to 23 nucleotides were analyzed.

The binding sites to the loci seed sequence were defined as a vsRNA candidate encoded by *Beta-CoV* genomes (Table 3). Among these nine potential vsRNAs, five (55%) were found in RNA structures belonging to ORF1a, while two (22%) were in ORF1b, and one (11% each) in both S and ORF3a. Next, upon a comparative analysis across the three hosts, it was discovered that more than one predicted vsRNA was shared between two hosts, indicating possibly a highly conserved RNA structure during viral evolution (Figure S4C). This finding was observed in SARS-CoV-2, intermediate animals (S2I-vsRNA-ORF1a-5p), and humans (S2H-vsRNA-ORF1a-5p) at genome positions ranging from 4153 to 4189. As these vsRNAs are similar in sequence and structure, we selected only one, modifying its nomenclature according to the hosts in which it is shared (S2I-H- vsRNA-ORF1a-5p).

**Figure 4:**
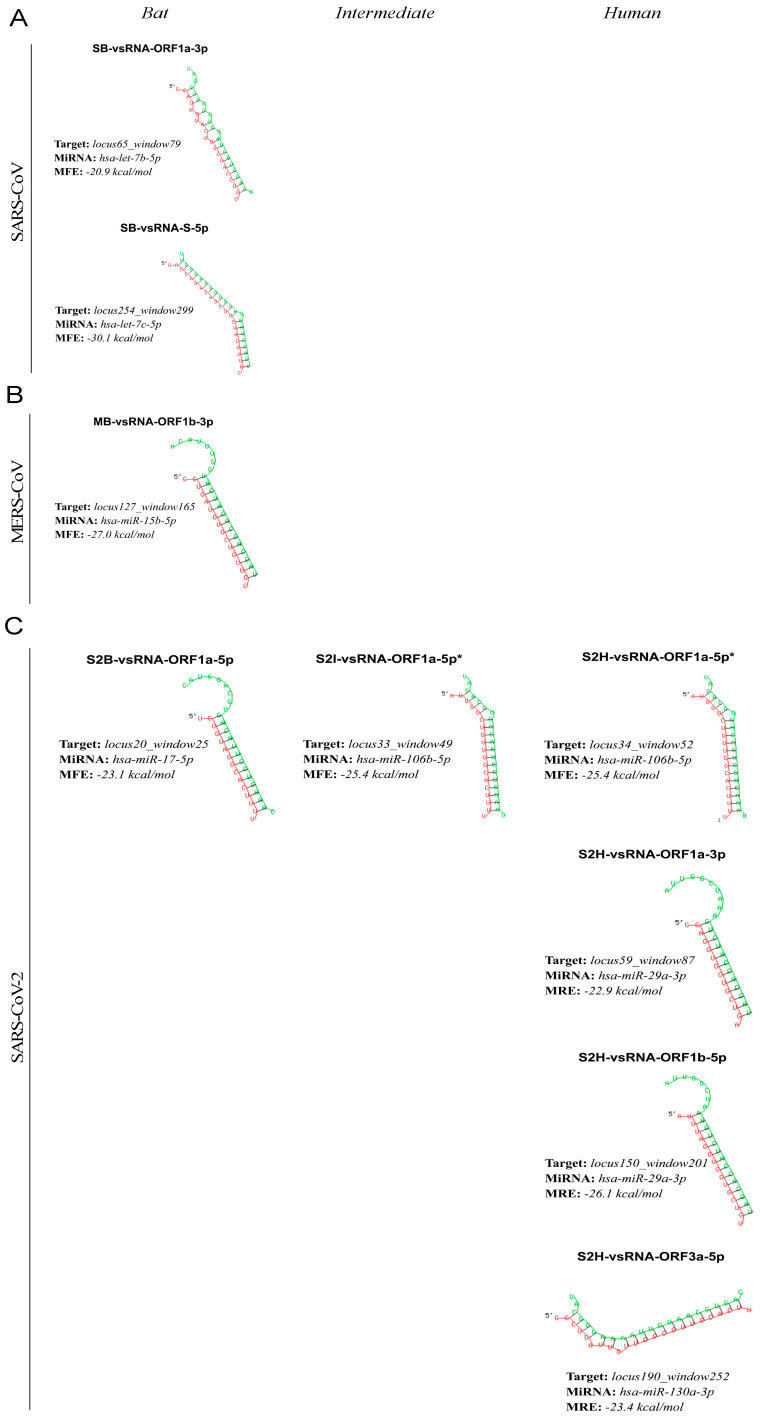
Putative illustration of vsRNA structures from the hsa-RNA:viralRNA interaction in *Beta-CoVs*. (**A**) For SARS-CoV, RNAhybrid, miRanda, and mirTarP agree with SB-vsRNA-ORF1a-3p and SB-vsRNA-S-5p detected in the viral genomes of bats. (**B**) Likewise, only MB-vsRNA-ORF1b-3p is predicted in bat viruses with matching by RNAhybrid, miRanda, and mirTarP in MERS-CoV. (**C**) Conversely, RNAhybrid, miRanda, and mirTarP agree with identifying vsRNAs in the three hosts for SARS-CoV-2. For bat-associated viruses, S2B-vsRNA-ORF1a-5p is detected, in intermediate animals, S2I-vsRNA-ORF1a-5p, shared with human viruses S2H-vsRNA-ORF1a-5p, along with S2H-vsRNA-ORF1a-3p, S2H-vsRNA-ORF1b-5p, and S2H-vsRNA-ORF3a-5p. Interactions show the target as the locus depicted by the sequence written in red (vsRNA), the hsa-miRNA shown in green, and the free energy MFE.

In more detail, RNA–RNA bindings in SARS-CoV and MERS-CoV carried two and one vsRNAs only in bats (SB-vsRNA-ORF1a-3p; SB-vsRNA-S-5p) and (MB-vsRNA-ORF1b-3p) with an MFE between −20.9 and −27.7 kcal/mol, respectively (Figure 4A,B). Conversely, SARS-CoV-2 showed vsRNAs across the three hosts: one in bat viruses (S2B-vsRNA-ORF1a-5p), one in intermediate animals (S2I-vsRNA-ORF1a-5p), and four in humans (S2H-vsRNA-ORF1a-5p; S2H-vsRNA-ORF1a-3p; S2H-vsRNA-ORF1b-3p; S2H-vsRNA-ORF3a-3p), where the MFE ranged from −22.1 to −26.1 kcal/mol (Figure 4C).

### 3.6. ORF1a Is a Crucial Viral Gene in Modulating the Host Transcriptome

First, we asked whether these nine potential vsRNAs had significant similarities with miRNAs from any organism. To this end, the miRBase database was used to search based on the miRNA seed sequence. Surprisingly, none of our predicted vsRNAs showed miRNAs associated with other organisms, which is a promising result (Table S5). In light of this, the following purpose was to understand what processes may affect this subset of *Beta-CoV* vsRNAs, assuming that they act as host gene regulators. Diana (MR-microT software) [54] and miRDB (Custom prediction) [45] tools with a score ≥ 70 were employed to obtain a reliable set of candidate human genes potentially targeted by these vsRNAs. A total of 1998 human genes were predicted as possible vsRNA targets for the three viruses. Despite SARS-CoV having two vsRNA candidates, it registered the most significant number of targets compared to the other viruses, resulting in 1076 ranging from 102 (SB-vsRNA-S-5p) to 974 (SB-vsRNA-ORF1a-3p), 538 being the average number of targets across the vsRNAs predicted (Figure S5A). Instead, SARS-CoV-2 with five potential vsRNAs scored 879 targets, between 82 (S2H-vsRNA-ORF1a-3p) and 393 (S2H-vsRNA-ORF1b-3p) averaging 175.8 (Figure S5C). As MERS-CoV only has one vsRNA, 43 targets were predicted (Figure S5B). The full details of each vsRNA targeting human genes can be consulted depending on the virus: SARS-CoV (Table S6), MERS-CoV (Table S7), and SARS-CoV-2 (Table S8). 

**Figure 5:**
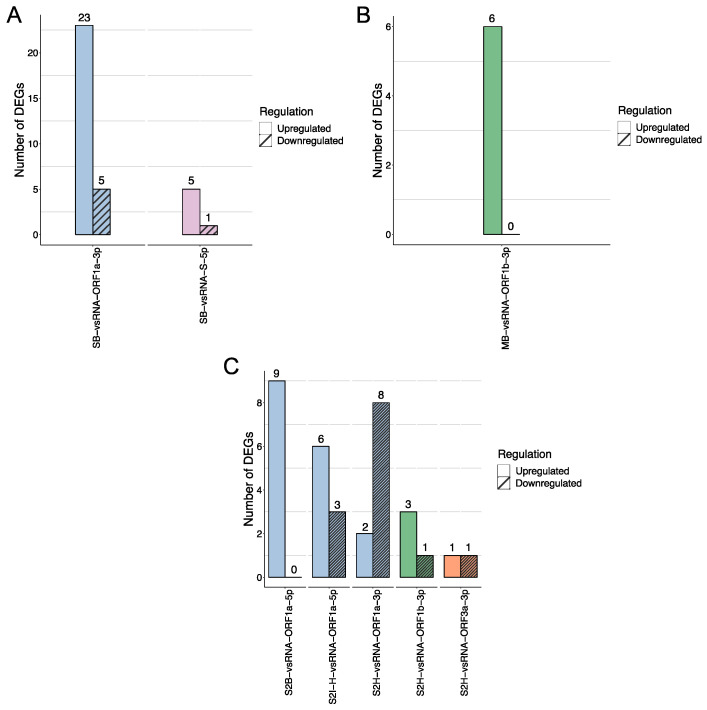
*Beta-CoV* vsRNAs targeting DEGs upon human lung infection. Prominent DEGs were possibly silenced up or down for each vsRNA according to the predicted targets. (**A**) vsRNAs encoded by SARS-CoV are targeted to 34 DEGs (28 up- and 6 downregulated); (**B**) MERS-CoV only shows 6 DEGs that are upregulated; and (**C**) SARS-CoV-2 also reports 34 DEGs (21 up- and 13 downregulated). Each vsRNA is colored given the detected ORF (ORF1a: blue; ORF1b: green; S: purple; ORF3a: orange).

Considering that our assumption relies on RNA–RNA interactions acting in a vsRNA fashion, capable of exploiting host machinery as miRNA-like molecules to regulate host transcriptional reprogramming [20], we selected the targets identified and cross-validated them with the list of DEGs for each *Beta-CoV* vsRNA. Out of the 1998 putative targets, 74 (55 up- and 19 downregulated) were detected as DEGs targeted by vsRNAs that were likely regulated upon infection (Figure 5). In particular, 34 DEGs (46% each) were identified for SARS-CoV (two vsRNAs: 28 up- and 6 downregulated) and SARS-CoV-2 (five vsRNAs: 21 up- and 13 downregulated) (Figure 5A,C). Conversely, the only MERS-CoV vsRNA detected six DEGs (14%) that were upregulated (Figure 5B). Interestingly, most of the vsRNAs targeting DEGs were detected in ORF1a, 56 (41%); followed by ORF1b, 10 (7.4%); S, 6 (4.4%); and ORF3a, 2 (1.5%). In addition, 40 (72%) and 16 (84%) of up- and downregulated DEGs were in ORF1a, suggesting a critical viral gene modulating the host transcriptome.

### 3.7. vsRNAs Are Probably Shutting Down Genes Associated with Neurotrophin Signaling Impairment upon Infection

**Figure 6:**
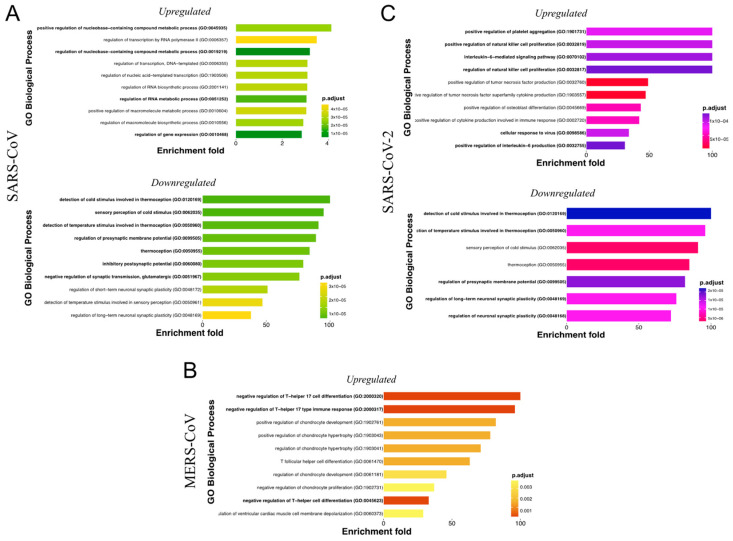
Functional enrichment analysis of DEGs regulated by *Beta-CoV* vsRNAs. Over-representation analysis for gene ontology showing biological process with adjusted *p*-value < 0.05 using a Fisher’s test for up- and downregulated DEGs in (**A**) SARS-CoV, (**B**) MERS-CoV, and (**C**) SARS-CoV-2. Each horizontal bar depicts a term and is grouped based on the ontology hierarchy, where the term in bold is the most relevant.

An ORA was performed using GO terms with the PANTHER online tool to better understand the functional alterations induced by up- and downregulated DEGs following lung epithelium infection with *Beta-CoVs*. Figure 6A shows the DEGs regulated by SARS-CoV vsRNAs. It is appreciated that vsRNA candidates of bat-associated viruses upregulate important BP terms in human lung cells, including regulation of the nucleobase-containing compound metabolic process (GO:0019219) and regulation of the RNA metabolic process (GO:0051252), mainly triggered by the increased expression level of *KLF4* (Diana: 92; miRDB: 75; log_2_FC: 4.46) and *PPARGC1A* (Diana: 90; miRDB: 75; log_2_FC: 3.20). Interestingly, 83% of downregulated DEGs were targeted by ORF1a vsRNAs, highlighting the BP terms associated with the regulation of presynaptic membrane potential (GO:0099505) by *GRIK2* (Diana: 100; miRDB: 82; log_2_FC: −1.92). On the other hand, the most enriched Reactome pathways for up- and downregulated DEGs were related to a generic transcription pathway (R-HSA-212436) and retrograde neurotrophin signaling (R-HSA-177504), respectively (Table S9).

Regarding MERS-CoV (Figure 6B), the enrichment analysis of bat vsRNA showed that several enriched BP terms for upregulated DEGs were associated with the regulation of T-helper 17 cell differentiation (GO:2000320), involving DEGs such as *SMAD7* (Diana: 76 miRDB: 77; log_2_FC: 4.62), and *RC3H1* (Diana: 78; miRDB: 77; log_2_FC: 3.05). In addition, Reactome pathway analyses hinted that TGF-beta receptor signaling activates SMADs (R-HSA-2173789) (Table S9).

Unlike SARS-CoV and MERS-CoV, most of the SARS-CoV-2 vsRNAs were predicted in human virus genomes, allowing a closer approximation of how infection may alter gene expression in human epithelial cells (Figure 6C). For instance, upregulated DEGs were enriched mainly by BP terms such as the regulation of natural killer cell proliferation (GO:0032819), interleukin-6-mediated signaling pathway (GO:0070102), regulation of tumor necrosis factor production (GO:0032760), and regulation of cytokine production involved in immune response (GO:0002720). DEGs engaged in these processes were highly over-expressed, namely, the cytokine family (*CXCL8* [Diana: 97; miRDB: 97; log_2_FC: 2.31], and *CXCL11* [Diana: 96; miRDB: 97; log_2_FC: 8.09]), interleukins (*IL-6* [Diana: 100; miRDB: 99; log_2_FC: 5.96]), and proinflammatory genes (*JAK2* [Diana: 85; miRDB: 90; log_2_FC: 2.95], *STAT2* [Diana: 97; miRDB: 97; log_2_FC: 2.88], and *MXD1* [Diana: 99; miRDB: 99; log_2_FC: 2.91]). Although these BP terms are already widely known in human SARS-CoV-2 infection [56,57,58], the most remarkable findings concern downregulated DEGs, where ORF1a vsRNAs targeted 78%. Similar to SARS-CoV, these DEGs were involved in the regulation of presynaptic membrane potential (GO:0099505), which together with *GRIK2* (Diana: 97; miRDB: 99; log_2_FC: −2.04), also participated *L1CAM* (Diana: 96; miRDB: 73; log_2_FC: −1.75) and *NEFL* (Diana: 98; miRDB: 98; log_2_FC: −1.72). Taking into consideration the upregulated DEGs, the most enriched Reactome pathways were cytokine signaling in the immune system (R-HSA-1280215), interferon signaling (R-HSA-913531), and SARS-CoV-2 activates/modulates innate and adaptive immune responses (R-HSA-9705671). At the same time, those downregulated were involved in retrograde neurotrophin signaling (R-HSA-177504) (Table S9).

## 4. Discussion

Since a picture is emerging in which various DNA viruses have been documented to encode v-miRNAs and undergo transcription and biogenesis similar to host miRNAs [59], whether RNA–RNA interactions of RNA viruses might function as putative vsRNAs has been a matter of controversy for several years. Nevertheless, several reports have demonstrated the functional identification v-miRNAs encoded by nuclear RNA viruses like retroviruses, which may benefit from host miRNA biogenesis machinery [21,22,23,60], as they have a DNA replication intermediate in their replication cycle and maintain long-term persistent infections [20,61]. Cytoplasmic RNA viruses do not confer this evolutionary advantage, as they may not access the nuclear enzyme Drosha needed for v-miRNA processing [62]. However, a recent study has shown that the hepatitis A virus may encode two novel functional v-miRNAs, hav-miR-1-5p and hav-miR-2-5p, which might hijack the host miRNA pathway [63]. Under these premises, we used RNA–RNA interactions of negative selection RNA structures to predict potential vsRNAs proposed as miRNA-like molecules, which might not fall into the more widely accepted category of miRNAs in the field [38].

In the wake of the SARS-CoV-2 pandemic, the discovery of vsRNA candidates in different *Beta-CoVs* has increasingly taken precedence. Unlike multiple reports that have shown computationally the presence of putative *Beta-CoV*-derived v-miRNAs (Table 2), our study employed RNA–RNA bindings of conserved RNA structures undergoing negative selection in the genomes of SARS-CoV, MERS-CoV, and SARS-CoV-2 circulating in different bat species, intermediate animals, and human hosts across the globe. Additionally, most studies predict v-miRNAs using the complete list of hsa-miRNAs from miRbase v22 [48] to be aligned against *Beta-CoV* genomes [64,65,66,67,68,69], and even in the Kyoto Encyclopedia of Genes and Genomes (KEGG) map pathways database [70], increasing the false positive rate of v-miRNAs considerably. Instead, we focused only on *Beta-CoVs* targeting hsa-microRNAs previously predicted upon complete validation by computational or experimental reports, coupled with sophisticated long-range RNA–RNA interactions from six highly cited prediction tools such as TargetScan [44], miRDB [45], and miRTarBase [46], integrated into miRWalk 3.0 [47] and corroborated with RNAhybrid [50], miRanda [51], and mirTarP [52], to refine the seed sequence homology of hsa-miRNAs with RNA structures under negative selection. More importantly, our results always are cross-validated with DEGs induced by human lung cells infected with *Beta-CoVs* for each RNA–RNA interaction.

Considering our RNA–RNA strategy, nine potential vsRNAs conserved in negatively selected RNA structures of *Beta-CoVs* are proposed. Among them, two vsRNAs are encoded by SARS-CoV circulating in bats (SB-vsRNA-ORF1a-3p; SB-vsRNA-S-5p), one vsRNA for MERS-CoV circulating in bats (MB-vsRNA-ORF1b-3p), and six vsRNAs for SARS-CoV-2 distributing in bats (S2B-vsRNA-ORF1a-5p), intermediate animals (S2I-vsRNA-ORF1a-5p), and humans (S2H-vsRNA-ORF1a-5p; S2H-vsRNA-ORF1a-3p; S2H-vsRNA-ORF1b-3p; S2H-vsRNA-ORF3a-3p). Remarkably, we report for the first time that two SARS-CoV-2 vsRNAs from intermediate animals (S2I-vsRNA-ORF1a-5p) and humans (S2H-vsRNA-ORF1a-5p) at positions 4153 to 4189 (ORF1a) match in both sequence and structure with an identical MFE, indicating the RNA structure is strongly under negative selection during viral evolution [9,11,71]. Efforts addressed to identify vsRNAs in the different *Beta-CoV* hosts start from scratch. Most are dedicated to human-associated SARS-CoV-2, given the context of the current clinical crisis. Nevertheless, a recent study has been reported for SARS-CoV, which falls into the intermediate host category. Morales and coworkers identified three vsRNAs in lung derived from SARS-CoV-infected mice using deep sequencing. This study demonstrates that vsRNA molecules derived from N protein and nonstructural protein 3 (nsp3) of ORF1a contribute to SARS-CoV lung pathogenesis, which is reduced by the antagonization of these vsRNAs [72]. There are no findings of possible vsRNA candidates concerning MERS-CoV, and SARS-CoV-2 is highlighted from infected human cells. For instance, a Northern blot assay-based study demonstrated that SARS-CoV-2 could encode miRNA-like vsRNA, namely vmiR-5p, from its ORF7a associated with the pathogenesis of the virus [73]. In addition, the deep sequencing of infected Calu-3 and Vero E6 cells showed that the SARS-CoV-2 N protein might encode vsRNAs v-miRNA-N-28612, v-miRNA-N-29094, and v-miRNA-N-29443 with a positive association with viral load in COVID-19 patients. More recently, Singh and coworkers identified a CoV2-miR-O7a as vsRNA in the ORF7a using small sequence RNAs from SARS-CoV-2-infected human A549-ACE2 cells, which have the potential to interfere with host transcripts involved in IFN signaling [74]. These studies were the stepping stones that paved the way for further exploring that SARS-CoV-2, a cytoplasmic RNA virus, might generate vsRNAs during infection.

Interestingly, it has been suggested that many RNA viruses contain “hotspots” that serve as sites for vsRNA production [62], whereby sequences surrounding hotspots might be expected to adopt stable hairpin-like structures predicted by RNAz within the conserved RNA structures [75,76]. This pattern was recently observed by comparing virally derived small RNAs in different SARS-CoV-2-infected samples that are prone to generate vsRNAs [77]. In our case, the vast majority of our *Beta-CoV* vsRNAs encoded in ORF1a at genome positions ranging from 4153 to 5544 seem to show possible “hotspots”, indicating that the nsp3 of ORF1a is deeply involved in vsRNA production [78,79]. Intriguingly, Fu and coworkers found a vsRNA in the 3′ arm of a hairpin within nsp3, namely miR-nsp3-3p, which appears to indicate the risk of occurrence of critical illness among COVID-19 patients [80]. Indeed, a computational study discovered the expression of eight putative vsRNAs in SARS-CoV-2-infected human lung cancer cells using deep learning. They also found vsRNAs in viral ORF1a that may regulate the host transcriptome upon infection [34]. These results indicate that the nsp3 of ORF1a appears to be a prominent hotspot to produce specific vsRNAs from bat virus transmission to humans. However, further functional studies are required to validate this result, especially for Omicron SARS-CoV-2 variants [81,82,83].

Regarding the function of potential vsRNAs, it is tempting to speculate that microRNA-like molecules might modulate host gene expression upon infection. A limitation of this study relies on RNA-seq data from the human lung cancer cell line (Calu-3) infected with SARS-CoV, MERS-CoV, and SARS-CoV-2, which assumes that the predicted vsRNAs in viruses isolated from bats and different animal species may, in part, also infect the human upper respiratory tract and promote their post-transcriptional regulation. Remarkably, among 74 DEGs, we found 55 up- and 19 downregulated DEGs upon infection according to the II and IM comparisons between the time points assessed. This may suggest that our vsRNA candidates would be produced either at different stages of the viral replication cycle or depending on the viral load in the cell, triggering probably post-transcriptional gene silencing under particular conditions [84]. Regarding upregulated DEGs, it would be expected that vsRNAs encoded by bat-associated SARS-CoV increased the expression levels of *KLF4* and *PPARGC1A* in human lung epithelium, which are involved in the gene transcription pathway. Likewise, the unique vsRNA predicted in MERS-CoV bats appears to induce the differentiation of human T-helper 17 cells via *SMAD7* and *RC3H1.*

In contrast, as most SARS-CoV-2 vsRNAs were detected in human viruses, we observed an over-expression of critical genes associated with the acute respiratory distress syndrome (ARDS) seen in COVID-19 patients, such as cytokines (*CXCL8* and *CXCL11*) [85], interleukins (IL-6) [86], and proinflammatory genes (*JAK2*, *STAT2,* and *MXD1*) [87]. Nevertheless, the premise of this study is that 83% and 73% of downregulated DEGs are targeted by vsRNAs encoded by the nsp3 of ORF1a, produced in bat SARS-CoV and human SARS-CoV-2 genomes, respectively. Most importantly, these vsRNAs appear to be shutting down genes implicated in the mechanisms regulating synaptic function, such as *GRIK2*, *L1CAM,* and *NEFL*, which are largely responsible for the neurotrophin signaling impairment of SARS-CoV-2 [88,89]. Although the role of SARS-CoV-2 in neurotrophins is an emerging trend, it has been reported that patients positive for SARS-CoV-2 have increased levels of brain-derived neurotrophic factor (BDNF) [90,91], a pivotal regulator of synaptic neuroplasticity, where its dysregulation might mediate in the pathogenesis of diverse neurological diseases such as Alzheimer’s disease (AD) [92]. To the best of our knowledge, we report the first potential RNA–RNA interactions capable of acting as vsRNAs linked to SARS-CoV-2 neuropathologies. Although recent publications show experimental evidence that there exists an interphase of RNA–RNA interactions on A549, Vero E6, and A549hACE2 cells infected with SARS-CoV-2, their findings cannot draw conclusions in other cell types [93,94]. Due to the limitation of our survey, we claim that further experimental studies are warranted to confirm the legitimate identity of these putative vsRNAs detected in Calu-3 cells infected with SARS-CoV-2 to understand their function and to implement strategies to be used as potential biomarkers to interrupt the course of SARS-CoV-2-induced neurological manifestations.

## 5. Conclusions

We implemented a novel computational approach to predict nine potential vsRNAs from negatively selected RNA structures of SARS-CoV, MERS-CoV, and SARS-CoV-2 circulating in bats, intermediate animals, and humans, coupled with a complete review of *Beta-CoVs* targeting hsa-miRNAs and sophisticated long-range RNA–RNA interactions as well as validation using RNA-seq data. We found that most of our vsRNA candidates are encoded by the nsp3 of ORF1a, which transcriptionally dysregulated 74 DEGs in human epithelial cells (Calu-3). Among them, 55 upregulate molecular mechanisms underlying ARDS, whereas 19 downregulated DEGs might be implicated in neurotrophin signaling impairment. However, further experimental studies are needed to consider our predicted vsRNAs as potential biomarkers against *Beta-CoV* pathologies.

## Figures and Tables

**Table 1:** Comparison of the number of loci bearing conserved RNA structures under selective pressures and those with negative selection, which shows the viral genome coverage.

Virus	Host	Total of Loci under Selection Pressures	Total of Loci under Negative Selection	^1^ Genome Coverage (%)
SARS-CoV	Bat	46	32	12.91
	Intermediate	62	59	23.85
	Human	72	68	27.47
MERS-CoV	Bat	85	78	31.17
	Intermediate	86	70	27.90
	Human	83	65	25.90
SARS-CoV-2	Bat	40	36	14.51
	Intermediate	69	45	18.16
	Human	76	52	20.92

^1^ Genome coverage percentage was calculated by multiplying the total number of nucleotides of all predicted loci by 100 and then dividing the viral genome length of a given host, shown in Figure S1.

**Table 2:** Number of hsa-miRNAs targeting *Beta-CoVs* supported by either computational or experimental studies.

Virus	Number of hsa-miRNAs	Detection Method	Number of Articles	Date Range for Publication Search
SARS-CoV	36	Computational	1	2002–2022
		Experimental	2	
		Computational/Experimental	1	
MERS-CoV	70	Computational	3	2012–2022
		Experimental	2	
SARS-CoV-2	150	Computational	10	2019–2022
		Experimental	7	
		Computational/Experimental	5	

**Table 3:** Overview of potential vsRNA candidates identified within conserved RNA structures with negative selection in *Beta-CoVs*.

Virus	Host	vsRNA Name	vsRNA Sequence	vsRNA Length	Genome Position	Strand	ORF	RNAhybrid MFE
SARS-CoV	Bat	SB-vsRNA-ORF1a-3p	GCAUUUUACGUGCUACCUUC	20	4250–4270	Forward	ORF1a	−20.9
	Bat	SB-vsRNA-S-5p	UACCAUACAGCUUCUACUUUAC	22	23,434–23,456	Forward	S	−27.7
MERS-CoV	Bat	MB-vsRNA-ORF1b-3p	GAGGUGAUGUGCUGUUGG	18	15,575–15,593	Forward	ORF1b	−27.0
SARS-CoV-2	Bat	S2B-vsRNA-ORF1a-5p	UUAUCUGUAGGCACUUUU	18	4212–4230	Reverse	ORF1a	−23.1
	Intermediate	S2I-vsRNA-ORF1a-5p *	AUUGUCUGUUGGCACUUUU	19	4170–4189	Reverse	ORF1a	−25.4
	Human	S2H-vsRNA-ORF1a-5p *	AUUGUCUGUUGGCACUUUU	19	4153–4172	Reverse	ORF1a	−25.4
	Human	S2H-vsRNA-ORF1a-3p	CUGAGCAGGUGGUGCUGA	18	5526–5544	Reverse	ORF1a	−22.9
	Human	S2H-vsRNA-ORF1b-3p	CAAUUUAGGUGGUGCUGU	18	19,443–19,461	Forward	ORF1b	−26.1
	Human	S2H-vsRNA-ORF3a-3p	GGCUUAUUGUUGGCGUUGCACUU	23	25,459–25,482	Forward	ORF3a	−23.4

* vsRNAs showing similar sequence, structure, and MFE.

## Data Availability

The data presented in this study are openly available in FigShare at http://doi.org/10.6084/m9.figshare.21312270, reference number 21312270 (will be published upon publication of the article).

## Supplementary Materials

The following supporting information can be downloaded at https://www.mdpi.com/article/10.3390/v15081647/s1, Figure S1: Flowchart showing approach to data collection, curation process, and range of sequence lengths for the three *Beta-CoVs* used in the study by [39], Figure S2: Volcano plots showing the differential expression profiling of mock cultures of Calu-3 cells infected with *Beta-CoVs* between the final and initial time (MM), Figure S3: Potential common miRNA:virusRNA pairings predicted by RNAhybrid, miRanda, and mirTarP in *Beta-CoVs*, Figure S4: Comparison of vsRNAs encoded by *Beta-CoVs* across hosts of bats, intermediate animals, and humans, Figure S5: Number of predicted targets in the human genome for each vsRNA candidate in the three *Beta-CoVs*, Table S1: DEGs of *Beta-CoVs* using a |log_2_FC| > 1.5 and FDR < 0.05, Table S2: Full list of hsa-miRNAs targeting *Beta-CoVs* highly supported by either computational or experimental studies [35,66,67,68,69,95,96,97,98,99,100,101,102,103,104,105,106,107,108,109,110,111,112,113,114,115,116,117,118,119,120,121], Table S3: Number of hsa-miRNA:3′UTR interactions resulting from RNAhybrid, miRanda, and mirTarP overlaps, Table S4: Number of hsa-miRNA:viralRNA interactions resulting from RNAhybrid, miRanda, and mirTarP overlaps, Table S5: Repertoire of identified vsRNAs in *Beta-CoV* genomes using RNA–RNA interactions, Table S6: Number of targets identified for SARS-CoV by vsRNAs, Table S7: Number of targets identified for MERS-CoV by vsRNAs, Table S8: Number of targets identified for SARS-CoV-2 by vsRNAs, Table S9: Functional enrichment of DEGs that are possibly silenced by vsRNAs identified in *Beta-CoVs.*
