# Supplementary material for: Computational Prediction of RNA–RNA Interactions between Small RNA Tracks from Betacoronavirus Nonstructural Protein 3 and Neurotrophin Genes during Infection of an Epithelial Lung Cancer Cell Line: Potential Role of Novel Small Regulatory RNA
Source: Viruses. 2023 Jul 28;15(8):1647. doi: 10.3390/v15081647 (PMC10458423; doi:10.3390/v15081647)
Supplement: Supplementary file 1 [file viruses-15-01647-s001.zip › Supplementary_Material/Supplementary_Figures.pdf]

viruses

Supplementary Figures

Computational Prediction of RNA–RNA Interactions between Small RNA Tracks from *Betacoronavirus* Nonstructural Protein 3 and Neurotrophin Genes during Infection of an Epithelial Lung Cancer Cell Line: Potential Role of Novel Small Regulatory RNA

Alexis Felipe Rojas-Cruz [1,2], Clara Isabel Bermúdez-Santana [1,2] *

[1] Theoretical and Computational RNomics Group, Department of Biology, Faculty of Sciences, National University of Colombia, Bogotá 111321, Colombia; alrojascr@unal.edu.co
[2] Center of Excellence in Scientific Computing, National University of Colombia, Bogotá 111321, Colombia
* Correspondence: cibermudezs@unal.edu.co

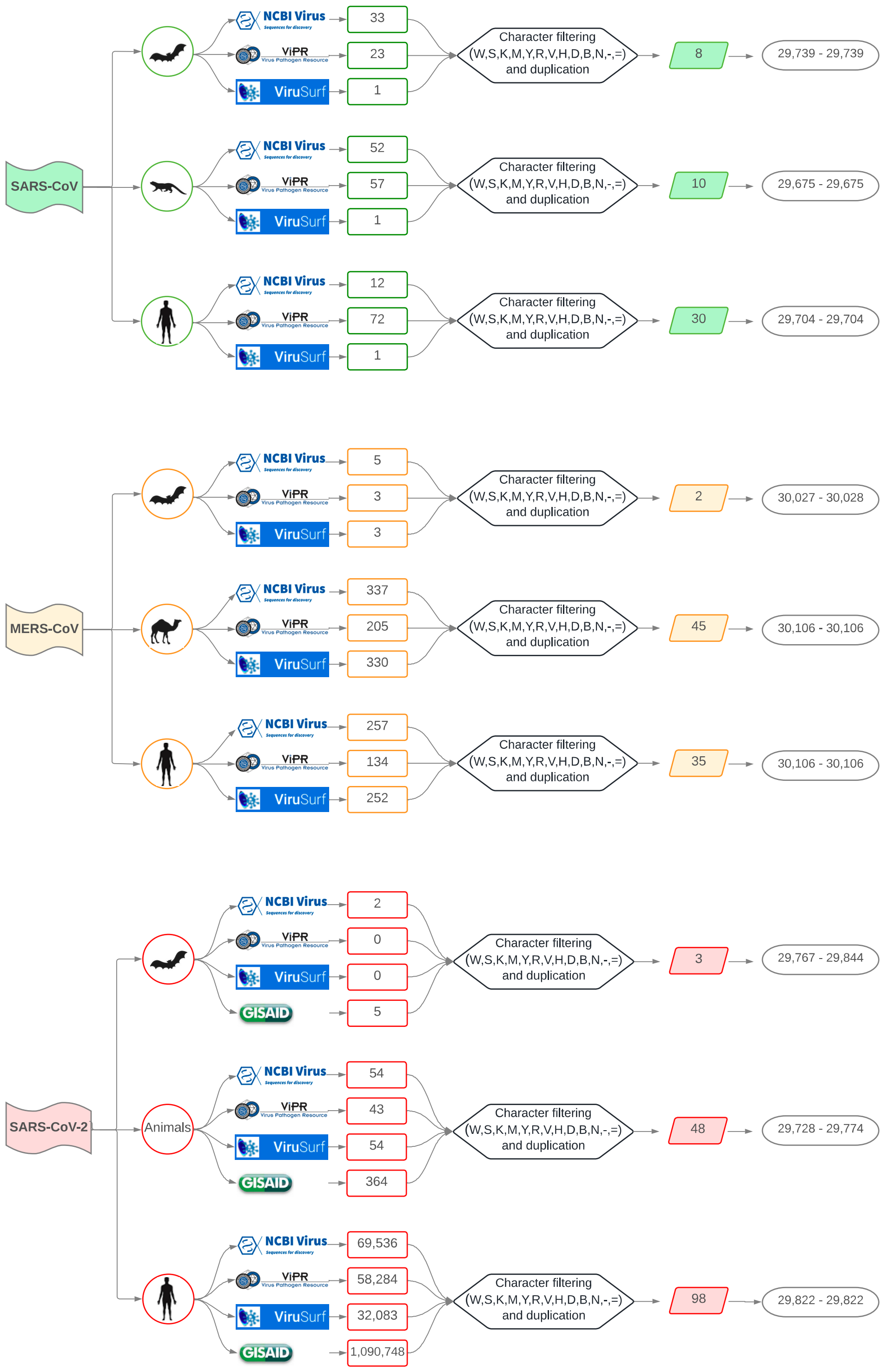

Figure S1. Flowchart showing approach to data collection, curation process and range of sequence lengths for the three *Beta-CoVs* used in the study by [39].

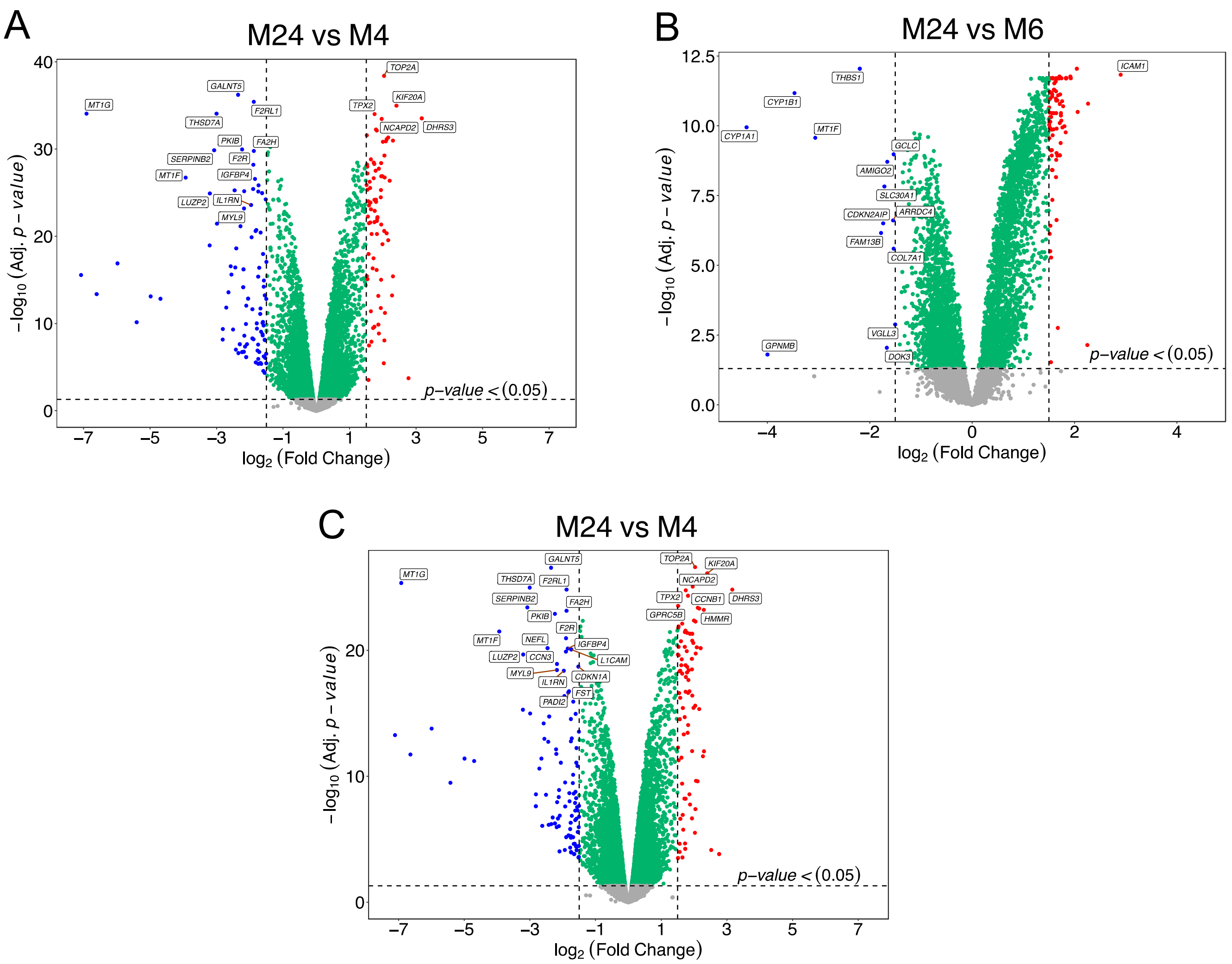

Figure S2. Volcano plots showing the differential expression profiling of mock cultures of Calu-3 cells infected with *Beta-CoVs* between the final and initial time (MM). Vertical lines denote an absolute value of |log2FC | > 1.5 versus -log10 (Adj. *p-value* < 0.05) in **(A)** SARS-CoV; **(B)** MERS-CoV; and **(C)** SARS-CoV-2. Upregulated DEGs are shown as red dots and downregulated DEGs as blue dots.

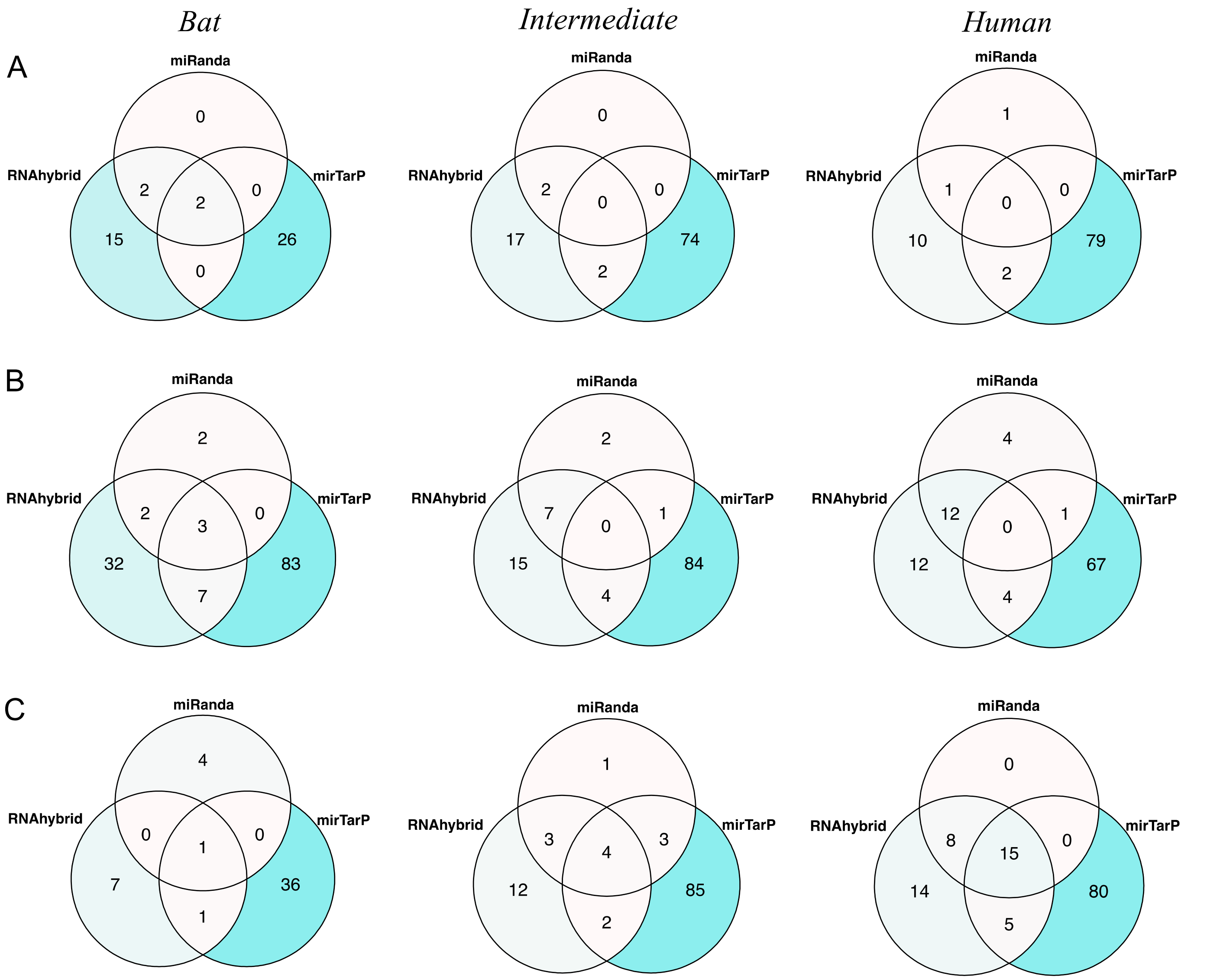

Figure S3. Potential common miRNAs:virusRNAs pairings predicted by RNAhybrid, miRanda and mirTarP in *Beta-CoVs*. **(A)** There are 2 interaction pairs in SARS-CoV; **(B)** 3 in MERS-CoV; and **(C)** 20 in SARS-CoV-2. Venn diagrams sections are colored based on the number of pairing predictions.

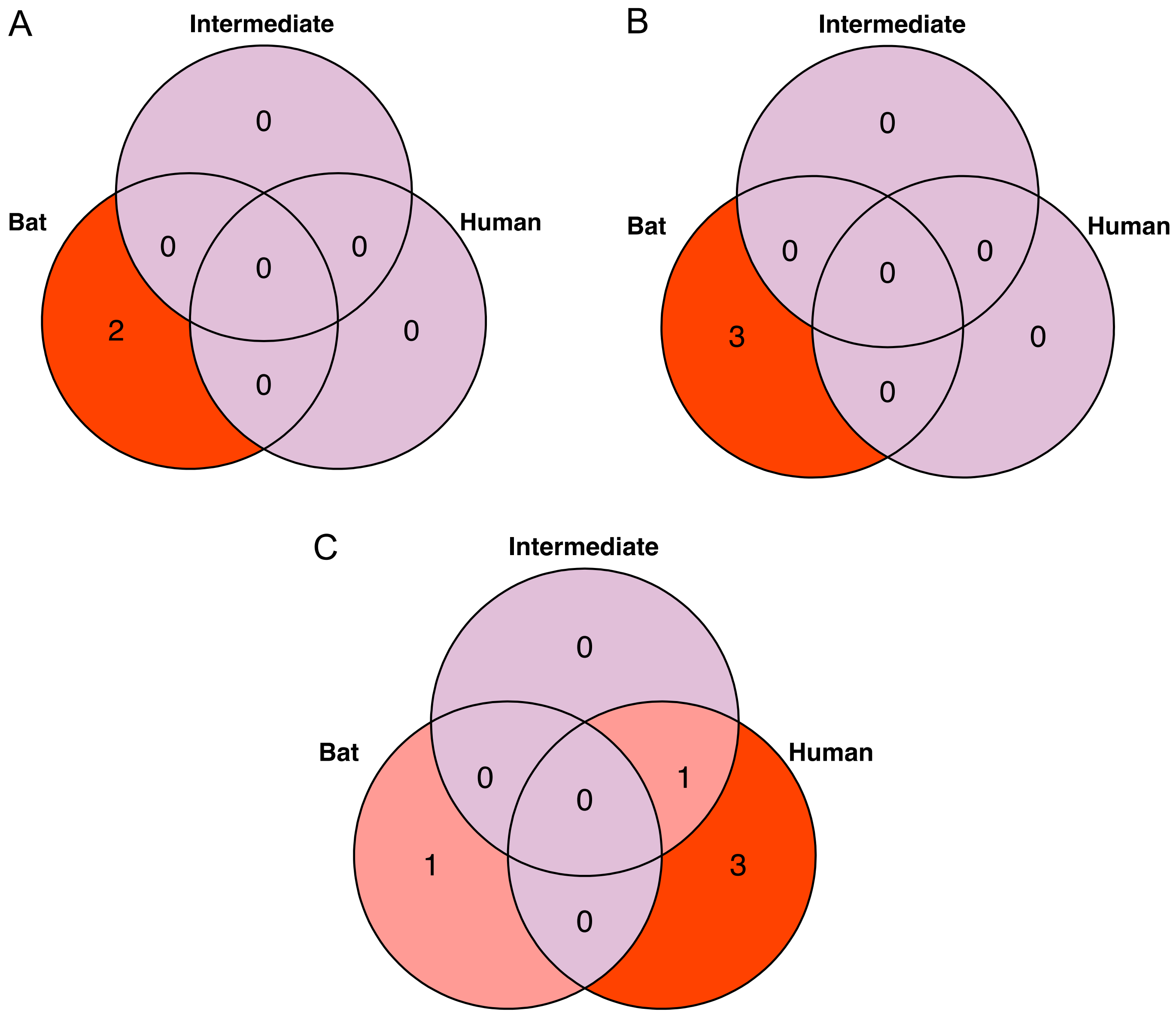

Figure S4. Comparison of vsRNAs encoded by *Beta-CoVs* across hosts of bats, intermediate animals, and humans. Venn diagrams show that 2 vsRNAs in **(A)** SARS-CoV, and 3 in **(B)** MERS-CoV are exclusive to bats, while 6 in **(C)** SARS-CoV-2 are observed in all three hosts, including 1 for bats, 1 for intermediate animals and 4 for humans.

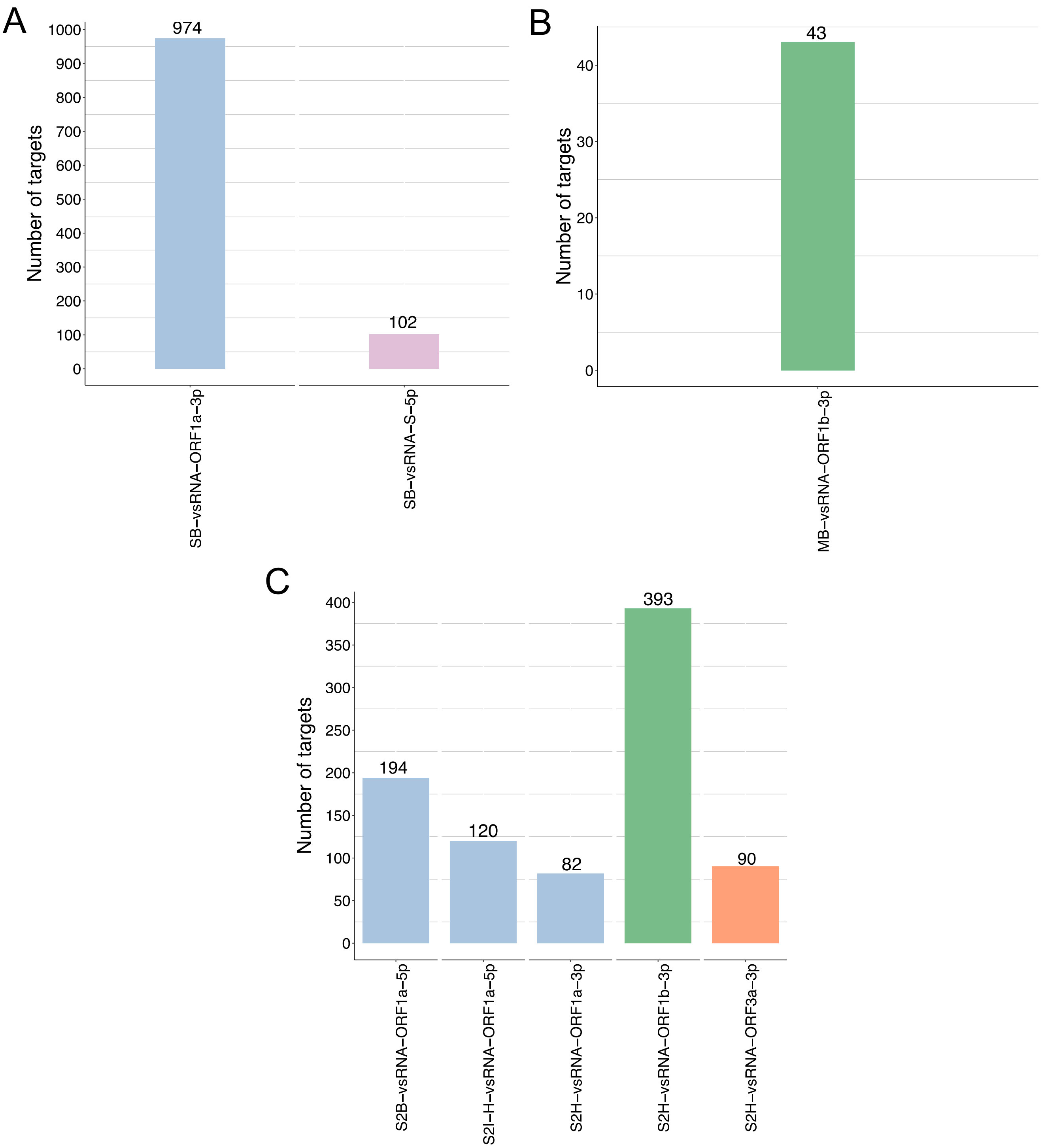

Figure S5. Number of predicted targets in the human genome for each vsRNA candidate in the three *Beta-CoVs*. **(A)** SARS-CoV encoded 2 vsRNAs registering 1076 targets; **(B)** MERS-CoV encoded 1 vsRNA showing 43 targets; and **(C)** SARS-CoV-2 with 5 vsRNAs obtained 879 human genes. Each vsRNA is colored given the detected ORF.
