# Supplementary material for: Computational Prediction of RNA–RNA Interactions between Small RNA Tracks from Betacoronavirus Nonstructural Protein 3 and Neurotrophin Genes during Infection of an Epithelial Lung Cancer Cell Line: Potential Role of Novel Small Regulatory RNA
Source: Viruses. 2023 Jul 28;15(8):1647. doi: 10.3390/v15081647 (PMC10458423; doi:10.3390/v15081647)
Supplement: Supplementary file 1 [file viruses-15-01647-s001.zip › Supplementary_Material/Table_S2.pdf]

Table S2. Full list of hsa-miRNAs targeting *Beta-CoVs* highly supported by either computational or experimental studies.

Virus	hsa-miRNA	Detection method	Year	Reference
SARS-CoV	hsa-let-7i-5p	Computational	2020	[1]
	hsa-let-7b-5p	Computational	2020	[1]
	hsa-let-7c-5p	Computational	2020	[1]
	hsa-let-7e-5p	Computational	2020	[1]
	hsa-let-7g-5p	Computational	2020	[1]
	hsa-miR-1202	Computational	2020	[1]
	hsa-miR-105-3p	Computational	2020	[1]
	hsa-miR-1224-5p	Computational	2020	[1]
	hsa-miR-4458	Computational	2020	[1]
	hsa-let-7f-5p	Computational	2020	[1]
	hsa-miR-1205	Computational	2020	[1]
	hsa-miR-1184	Computational	2020	[1]
	hsa-miR-1183	Computational	2020	[1]
	hsa-miR-103a-2-5p	Computational	2020	[1]
	hsa-miR-1208	Computational	2020	[1]
	hsa-miR-4500	Computational	2020	[1]
	hsa-miR-101-3p.2	Computational	2020	[1]
	hsa-let-7a-2-3p	Computational	2020	[1]
	hsa-miR-98-5p	Computational	2020	[1]
	hsa-let-7i-3p	Computational	2020	[1]
	hsa-let-7g-3p	Computational	2020	[1]
	hsa-miR-1197	Computational	2020	[1]
	hsa-miR-101-3p.1	Computational	2020	[1]
	hsa-miR-1199-5p	Computational	2020	[1]
	hsa-miR-103b	Computational	2020	[1]
	hsa-miR-10b-5p	Computational	2020	[1]
	hsa-miR-1178-3p	Computational	2020	[1]
	hsa-let-7d-5p	Computational	2020	[1]
	hsa-miR-17	Computational/Experimental	2009	[2]
	hsa-miR-574-5p	Computational/Experimental	2009	[2]
	hsa-miR-214	Computational/Experimental	2009	[2]
	hsa-miR-223	Computational/Experimental	2009	[2]
	hsa-miR-98	Computational/Experimental	2009	[2]
	hsa-miR-200c-3p	Experimental	2017	[3]
	hsa-miR-155-3p	Experimental	2021	[4]
	hsa-let-7a-3p	Experimental	2021	[4]
MERS-CoV	hsa-miR-15a-5p	Experimental	2020	[5]
	hsa-miR-15b-5p	Experimental	2020	[5]
	hsa-miR-195-5p	Experimental	2020	[5]
	hsa-miR-16-5p	Experimental	2020	[5]
	hsa-miR-196a-1-3p	Experimental	2020	[5]
	hsa-miR-16-1-3p	Experimental	2020	[6]
	hsa-miR-26a-1-3p	Experimental	2020	[6]

hsa-miR-425-5p	Experimental	2020	[6]
hsa-miR-500b-5p	Experimental	2020	[6]
hsa-miR-627-5p	Experimental	2020	[6]
hsa-miR-1257	Experimental	2020	[6]
hsa-miR-1275	Experimental	2020	[6]
hsa-miR-2277-5p	Experimental	2020	[6]
hsa-miR-2392	Experimental	2020	[6]
hsa-miR-4448	Experimental	2020	[6]
hsa-miR-4455	Experimental	2020	[6]
hsa-miR-4521	Experimental	2020	[6]
hsa-miR-6807-5p	Experimental	2020	[6]
hsa-miR-6847-3p	Experimental	2020	[6]
hsa-miR-329-5p	Experimental	2020	[6]
hsa-miR-539-5p	Experimental	2020	[6]
hsa-miR-619-5p	Experimental	2020	[6]
hsa-miR-762	Experimental	2020	[6]
hsa-miR-6836-5p	Experimental	2020	[6]
hsa-miR-628-5p	Computational	2014	[7]
hsa-miR-332-3p	Computational	2014	[7]
hsa-miR-6804-3p	Computational	2014	[7]
hsa-miR-4289	Computational	2014	[7]
hsa-miR-208a-3p	Computational	2014	[7]
hsa-miR-510-3p	Computational	2014	[7]
hsa-miR-18a-3p	Computational	2014	[7]
hsa-miR-329-3p	Computational	2014	[7]
hsa-miR-548ax	Computational	2014	[7]
hsa-miR-3934-5p	Computational	2014	[7]
hsa-miR-4474-5p	Computational	2014	[7]
hsa-miR-7974	Computational	2014	[7]
hsa-miR-6865-5p	Computational	2014	[7]
hsa-miR-342-3p	Computational	2014	[7]
hsa-let-7i-5p	Computational	2020	[1]
hsa-let-7c-5p	Computational	2020	[1]
hsa-let-7e-5p	Computational	2020	[1]
hsa-let-7g-5p	Computational	2020	[1]
hsa-let-7b-5p	Computational	2020	[1]
hsa-miR-1202	Computational	2020	[1]
hsa-let-7d-5p	Computational	2020	[1]
hsa-let-7f-5p	Computational	2020	[1]
hsa-miR-98-5p	Computational	2020	[1]
hsa-miR-1224-5p	Computational	2020	[1]
hsa-miR-1184	Computational	2020	[1]
hsa-miR-105-3p	Computational	2020	[1]
hsa-miR-4458	Computational	2020	[1]
hsa-miR-1183	Computational	2020	[1]
hsa-let-7a-2-3p	Computational	2020	[1]

	hsa-miR-103a-2-5p	Computational	2020	[1]
	hsa-miR-4500	Computational	2020	[1]
	hsa-miR-10a-5p	Computational	2020	[1]
	hsa-miR-101-5p	Computational	2020	[1]
	hsa-miR-10b-5p	Computational	2020	[1]
	hsa-miR-1185-2-3p	Computational	2020	[1]
	hsa-miR-103b	Computational	2020	[1]
	hsa-miR-1185-1-3p	Computational	2020	[1]
	hsa-miR-3180-3p	Computational	2021	[8]
	hsa-miR-3173-3p	Computational	2021	[8]
	hsa-miR-1247-3p	Computational	2021	[8]
	hsa-miR-127-5p	Computational	2021	[8]
	hsa-miR-376a-3p	Computational	2021	[8]
	hsa-miR-3663-3p	Computational	2021	[8]
	hsa-miR-622	Computational	2021	[8]
	hsa-miR-3180	Computational	2021	[8]
	hsa-miR-4660	Computational	2021	[8]
SARS-CoV-2	hsa-miR-155-3p	Computational	2020	[1]
	hsa-miR-139-5p	Computational	2020	[1]
	hsa-let-7a-3p	Computational	2020	[1]
	hsa-miR-135b-5p	Computational	2020	[1]
	hsa-miR-16-2-3p	Computational, Experimental	2020, 2022	[1], [9]
	hsa-miR-1275	Computational	2020	[1]
	hsa-miR-5047	Computational, Computational, Computational	2020, 2020, 2020	[1], [10], [11]
	hsa-miR-1301-3p	Computational, Computational, Computational	2020, 2020, 2020	[1], [10], [11]
	hsa-miR-125a-3p	Computational, Computational, Computational	2020, 2020, 2020	[1], [10], [11]
	hsa-miR-196a-5p	Computational, Computational, Computational	2020, 2020, 2020	[1], [10], [11]
	hsa-miR-19b-2-5p	Computational, Computational, Computational	2020, 2020, 2020	[1], [10], [11]
	hsa-miR-4758-5p	Computational, Computational, Computational	2020, 2020, 2020	[1], [10], [11]
	hsa-miR-141-3p	Computational, Computational, Computational	2020, 2020, 2020	[1], [10], [11]
	hsa-miR-1202	Computational, Computational, Computational	2020, 2020, 2020	[1], [10], [11]
	hsa-miR-19b-1-5p	Computational, Computational, Computational	2020, 2020, 2020	[1], [10], [11]
	hsa-miR-15b-3p	Computational, Computational, Computational	2020, 2020, 2020	[1], [10], [11]
	hsa-miR-153-5p	Computational, Computational, Computational	2020, 2020, 2020	[1], [10], [11]
	hsa-miR-219a	Computational/Experimental	2021	[12]
	hsa-miR-29a	Computational/Experimental	2021	[12]
	hsa-miR-15b	Computational/Experimental	2021	[12]
	hsa-miR-30c	Computational/Experimental	2021	[12]
	hsa-miR-378d	Computational/Experimental	2021	[12]

hsa-miR-146a-5p	Computational, Experimental, Experimental, Experimental, Experimental	2020, 2020, 2022, 2022, 2022	[13], [14], [9], [15], [16]
hsa-miR-21-5p	Computational, Experimental	2020, 2020	[13], [14]
hsa-miR-142-3p	Experimental, Computational/Experimental, Experimental	2020, 2022, 2022	[14], [17], [18]
hsa-miR-486-5p	Experimental, Computational/Experimental, Experimental	2020, 2022, 2022	[14], [17], [18]
hsa-miR-181a-2-3p	Experimental	2020	[14]
hsa-miR-6869-5p	Computational	2020	[19]
hsa-miR-16-5p	Computational, Experimental, Experimental, Computational	2020, 2022, 2022, 2022	[19], [9], [18], [20]
hsa-miR-15a-5p	Computational	2020	[19]
hsa-miR-15b-5p	Computational	2020	[19]
hsa-miR-195-5p	Computational	2020	[19]
hsa-miR-497-5p	Computational	2020	[19]
hsa-miR-21-3p	Computational	2020	[19]
hsa-miR-424-5p	Computational, Experimental	2020, 2022	[19], [9]
hsa-miR-29a-3p	Computational	2020	[19]
hsa-miR-342-5p	Computational	2021	[21]
hsa-miR-432-5p	Computational	2021	[21]
hsa-miR-98-5p	Computational/Experimental, Computational	2020, 2021	[22], [21]
hsa-miR-17-5p	Computational, Experimental	2021, 2022	[21], [15]
hsa-miR-3941	Computational/Experimental	2021	[23]
hsa-miR-128-1-5p	Computational/Experimental	2021	[23]
hsa-miR-365b-5p	Computational/Experimental	2021	[23]
hsa-miR-138-5p	Computational/Experimental	2021	[23]
hsa-miR-34a-5p	Experimental, Computational/Experimental	2020, 2021	[24], [25]
hsa-miR-429	Computational/Experimental	2021	[25]
hsa-miR-7-5p	Computational/Experimental	2021	[25]
hsa-miR-155-5p	Computational/Experimental, Experimental	2021	[25], [16]
hsa-miR-203a-3p	Computational/Experimental	2021	[25]
hsa-miR-1307-3p	Computational	2020	[26]
hsa-miR-449c-5p	Computational	2020	[26]
hsa-miR-221-3p	Experimental, Experimental	2020, 2022	[5], [9]
hsa-miR-140-3p	Experimental, Experimental	2020, 2022	[5], [9]
hsa-miR-422a	Experimental	2020	[5]
hsa-miR-200c	Experimental	2020	[27]
hsa-miR-146b-5p	Experimental	2022	[16]
hsa-miR-499	Experimental	2022	[16]
hsa-miR-335-5p	Experimental	2022	[9]
hsa-miR-27b-3p	Experimental	2022	[9]
hsa-miR-660-5p	Experimental	2022	[9]
hsa-miR-130a-3p	Experimental	2022	[9]
hsa-miR-362-3p	Experimental	2022	[9]

hsa-miR-425-5p	Experimental	2022	[9]
hsa-miR-101-3p	Experimental	2022	[9]
hsa-miR-376a-3p	Experimental	2022	[9]
hsa-miR-126-5p	Experimental	2022	[9]
hsa-miR-382-5p	Experimental	2022	[9]
hsa-miR-451a	Experimental, Computational/Experimental	2022, 2022	[9], [17]
hsa-miR-19a-3p	Experimental	2022	[9]
hsa-miR-19b-3p	Experimental	2022	[9]
hsa-miR-24-3p	Experimental	2022	[9]
hsa-miR-199a-3p	Experimental	2022	[9]
hsa-miR-584-5p	Experimental	2022	[9]
hsa-miR-532-3p	Experimental	2022	[9]
hsa-miR-25-3p	Experimental	2022	[9]
hsa-miR-99b-5p	Experimental	2022	[9]
hsa-miR-361-5p	Experimental	2022	[9]
hsa-miR-194-5p	Experimental	2022	[9]
hsa-miR-29c-3p	Experimental	2022	[9]
hsa-miR-33a-5p	Experimental	2022	[9]
hsa-miR-106b-5p	Experimental	2022	[9]
hsa-miR-126-3p	Experimental, Experimental	2022, 2022	[9], [15]
hsa-miR-502-3p	Experimental	2022	[9]
hsa-miR-186-5p	Experimental	2022	[9]
hsa-miR-130b-3p	Experimental	2022	[9]
hsa-miR-30a-5p	Experimental	2022	[9]
hsa-miR-27a-3p	Experimental	2022	[15]
hsa-miR-495-3p	Experimental	2022	[15]
hsa-miR-4665-5p	Experimental	2022	[28]
hsa-miR-3190-3p	Experimental	2022	[28]
hsa-miR-331-3p	Experimental	2022	[28]
hsa-miR-4525	Experimental	2022	[28]
hsa-miR-431-5p	Experimental	2022	[28]
hsa-miR-6721-5p	Experimental	2022	[28]
hsa-miR-4661-5p	Experimental	2022	[28]
hsa-miR-548a-3p	Experimental	2022	[28]
hsa-miR-4745-5p	Experimental	2022	[28]
hsa-miR-3976	Experimental	2022	[28]
hsa-miR-4488	Experimental	2022	[28]
hsa-miR-3150b-3p	Experimental	2022	[28]
hsa-miR-7704	Experimental	2022	[28]
hsa-miR-3168	Experimental	2022	[28]
hsa-miR-3065-3p	Computational/Experimental	2022	[17]
hsa-miR-93-5p	Computational/Experimental	2022	[17]
hsa-miR-3065-5p	Computational/Experimental	2022	[17]
hsa-miR-628-3p	Computational/Experimental	2022	[17]
hsa-let-7b-5p	Computational, Experimental	2020, 2022	[13], [18]

hsa-miR-486-3p	Experimental	2022	[18]
hsa-let-7a-5p	Experimental	2022	[18]
hsa-let-7i-5p	Experimental	2022	[18]
hsa-let-7f-5p	Experimental	2022	[18]
hsa-miR-223-3p	Experimental	2022	[18]
hsa-miR-144-3p	Experimental	2022	[18]
hsa-miR-15a-3p	Experimental	2022	[18]
hsa-miR-374a-3p	Experimental, Computational	2022, 2022	[18], [20]
hsa-miR-181c-5p	Experimental	2022	[18]
hsa-miR-374a-5p	Experimental, Computational	2022, 2022	[18], [20]
hsa-miR-1277-5p	Experimental	2022	[18]
hsa-miR-369-3p	Experimental	2022	[18]
hsa-miR-146a-3p	Experimental	2022	[18]
hsa-miR-556-3p	Experimental	2022	[18]
hsa-miR-16-1-3p	Experimental	2022	[18]
hsa-miR-193b-5p	Experimental	2022	[18]
hsa-miR-4669	Experimental	2022	[18]
hsa-miR-193a-5p	Experimental	2022	[18]
hsa-miR-4697-3p	Experimental	2022	[18]
hsa-miR-4516	Experimental	2022	[18]
hsa-miR-1228-3p	Experimental	2022	[18]
hsa-miR-3182	Experimental	2022	[18]
hsa-miR-150-3p	Experimental	2022	[18]
hsa-miR-885-3p	Experimental	2022	[18]
hsa-miR-34c-3p	Experimental	2022	[18]
hsa-miR-148a	Experimental	2022	[29]
hsa-miR-34c	Experimental	2022	[29]
hsa-miR-34b	Experimental	2022	[29]
hsa-miR-342	Experimental	2022	[29]
hsa-miR-16	Computational	2020	[13]
hsa-miR-26a-5p	Experimental	2022	[30]
hsa-miR-29b-3p	Experimental	2022	[30]
hsa-miR-421	Experimental	2022	[30]
hsa-miR-3909	Experimental	2022	[30]
hsa-miR-212–5p	Experimental	2022	[30]
hsa-miR-4677–3p	Experimental	2022	[30]
hsa-let-7f-1-3p	Computational	2022	[20]
hsa-miR-548d-3p	Computational	2022	[20]
hsa-miR-23b-3p	Computational	2022	[20]
hsa-miR-302c-5p	Computational	2022	[20]
hsa-miR-143-5p	Computational	2022	[31]
hsa-miR-570-5p	Computational	2022	[31]
hsa-miR-34c-5p	Computational	2020	[32]

References

1. Chow, J.T.-S.; Salmena, L. Prediction and Analysis of SARS-CoV-2-Targeting MicroRNA in Human Lung Epithelium. *Genes* **2020**, *11*, doi:10.3390/genes11091002.
2. Mallick, B.; Ghosh, Z.; Chakrabarti, J. MicroRNome Analysis Unravels the Molecular Basis of SARS Infection in Bronchoalveolar Stem Cells. *PLOS ONE* **2009**, *4*, e7837, doi:10.1371/journal.pone.0007837.
3. Liu, Q.; Du, J.; Yu, X.; Xu, J.; Huang, F.; Li, X.; Zhang, C.; Li, X.; Chang, J.; Shang, D.; et al. MiRNA-200c-3p Is Crucial in Acute Respiratory Distress Syndrome. *Cell Discov.* **2017**, *3*, 1–17, doi:10.1038/celldisc.2017.21.
4. Wyler, E.; Mösbauer, K.; Franke, V.; Diag, A.; Gottula, L.T.; Arsiè, R.; Klironomos, F.; Koppstein, D.; Hönzke, K.; Ayoub, S.; et al. Transcriptomic Profiling of SARS-CoV-2 Infected Human Cell Lines Identifies HSP90 as Target for COVID-19 Therapy. *iScience* **2021**, *24*, 102151, doi:10.1016/j.isci.2021.102151.
5. Kim, W.R.; Park, E.G.; Kang, K.-W.; Lee, S.-M.; Kim, B.; Kim, H.-S. Expression Analyses of MicroRNAs in Hamster Lung Tissues Infected by SARS-CoV-2. *Mol. Cells* **2020**, *43*, 953–963, doi:10.14348/molcells.2020.0177.
6. Zhang, X.; Chu, H.; Wen, L.; Shuai, H.; Yang, D.; Wang, Y.; Hou, Y.; Zhu, Z.; Yuan, S.; Yin, F.; et al. Competing Endogenous RNA Network Profiling Reveals Novel Host Dependency Factors Required for MERS-CoV Propagation. *Emerg. Microbes Infect.* **2020**, *9*, 733–746, doi:10.1080/22221751.2020.1738277.
7. Hasan, M.M.; Akter, R.; Ullah, M.S.; Abedin, M.J.; Ullah, G.M.A.; Hossain, M.Z. A Computational Approach for Predicting Role of Human MicroRNAs in MERS-CoV Genome. *Adv. Bioinforma.* **2014**, *2014*, e967946, doi:10.1155/2014/967946.
8. Wu, Y.-H.; Yeh, I.-J.; Phan, N.N.; Yen, M.-C.; Hung, J.-H.; Chiao, C.-C.; Chen, C.-F.; Sun, Z.; Hsu, H.-P.; Wang, C.-Y.; et al. Gene Signatures and Potential Therapeutic Targets of Middle East Respiratory Syndrome Coronavirus (MERS-CoV)-Infected Human Lung Adenocarcinoma Epithelial Cells. *J. Microbiol. Immunol. Infect.* **2021**, *54*, 845, doi:10.1016/j.jmii.2021.03.007.
9. Martínez-Fleta, P.; Vera-Tomé, P.; Jiménez-Fernández, M.; Requena, S.; Roy-Vallejo, E.; Sanz-García, A.; Lozano-Prieto, M.; López-Sanz, C.; Vara, A.; Lancho-Sánchez, Á.; et al. A Differential Signature of Circulating MiRNAs and Cytokines Between COVID-19 and Community-Acquired Pneumonia Uncovers Novel Physiopathological Mechanisms of COVID-19. *Front. Immunol.* **2022**, *12*.
10. Saçar Demirci, M.D.; Adan, A. Computational Analysis of MicroRNA-Mediated Interactions in SARS-CoV-2 Infection. *PeerJ* **2020**, *8*, doi:10.7717/peerj.9369.
11. Fulzele, S.; Sahay, B.; Yusufu, I.; Lee, T.J.; Sharma, A.; Kolhe, R.; Isales, C.M. COVID-19 Virulence in Aged Patients Might Be Impacted by the Host Cellular MicroRNAs Abundance/Profile. *Aging Dis.* **2020**, *11*, 509–522, doi:10.14336/AD.2020.0428.
12. Siniscalchi, C.; Di Palo, A.; Russo, A.; Potenza, N. Human MicroRNAs Interacting With SARS-CoV-2 RNA Sequences: Computational Analysis and Experimental Target Validation. *Front. Genet.* **2021**, *12*, 760, doi:10.3389/fgene.2021.678994.
13. Jafarinejad-Farsangi, S.; Jazi, M.M.; Rostamzadeh, F.; Hadizadeh, M. High Affinity of Host Human MicroRNAs to SARS-CoV-2 Genome: An in Silico Analysis. *Non-Coding RNA Res.* **2020**, *5*, 222–231, doi:10.1016/j.ncrna.2020.11.005.
14. Tang, H.; Gao, Y.; Li, Z.; Miao, Y.; Huang, Z.; Liu, X.; Xie, L.; Li, H.; Wen, W.; Zheng, Y.; et al. The Noncoding and Coding Transcriptional Landscape of the Peripheral Immune Response in Patients with COVID-19. *Clin. Transl. Med.* **2020**, *10*, e200, doi:10.1002/ctm2.200.
15. García-Hidalgo, M.C.; González, J.; Benítez, I.D.; Carmona, P.; Santisteve, S.; Pérez-Pons, M.; Moncusí-Moix, A.; Gort-Paniello, C.; Rodríguez-Jara, F.; Molinero, M.; et al. Identification of Circulating MicroRNA Profiles Associated with Pulmonary Function and Radiologic Features in Survivors of SARS-CoV-2-Induced ARDS. *Emerg. Microbes Infect.* **2022**, *11*, 1537–1549, doi:10.1080/22221751.2022.2081615.
16. Kassif-Lerner, R.; Zloto, K.; Rubin, N.; Asraf, K.; Doolman, R.; Paret, G.; Nevo-Caspi, Y. MiR-155: A Potential Biomarker for Predicting Mortality in COVID-19 Patients. *J. Pers. Med.* **2022**, *12*, 324, doi:10.3390/jpm12020324.
17. Farr, R.J.; Rootes, C.L.; Stenos, J.; Foo, C.H.; Cowled, C.; Stewart, C.R. Detection of SARS-CoV-2 Infection by MicroRNA Profiling of the Upper Respiratory Tract. *PLOS ONE* **2022**, *17*, e0265670, doi:10.1371/journal.pone.0265670.
18. Garcia-Giralt, N.; Du, J.; Marin-Corral, J.; Bódalo-Torruella, M.; Blasco-Hernando, F.; Muñoz-Bermúdez, R.; Clarós, M.; Nonell, L.; Perera-Bel, J.; Fernandez-González, M.; et al. Circulating MicroRNA Profiling Is Altered in the Acute Respiratory Distress Syndrome Related to SARS-CoV-2 Infection. *Sci. Rep.* **2022**, *12*, 6929, doi:10.1038/s41598-022-10738-3.
19. Pierce, J.B.; Simion, V.; Icli, B.; Pérez-Cremades, D.; Cheng, H.S.; Feinberg, M.W. Computational Analysis of Targeting SARS-CoV-2, Viral Entry Proteins ACE2 and TMPRSS2, and Interferon Genes by Host MicroRNAs. *Genes* **2020**, *11*, 1354, doi:10.3390/genes11111354.
20. Li, C.; Wang, R.; Wu, A.; Yuan, T.; Song, K.; Bai, Y.; Liu, X. SARS-COV-2 as Potential MicroRNA Sponge in COVID-19 Patients. *BMC Med. Genomics* **2022**, *15*, 94, doi:10.1186/s12920-022-01243-7.
21. Banaganapalli, B.; Al-Rayes, N.; Awan, Z.A.; Alsulaimany, F.A.; Alamri, A.S.; Elango, R.; Malik, M.Z.; Shaik, N.A. Multilevel Systems Biology Analysis of Lung Transcriptomics Data Identifies Key MiRNAs and Potential MiRNA Target Genes for SARS-CoV-2 Infection. *Comput. Biol. Med.* **2021**, *135*, 104570, doi:10.1016/j.compbiomed.2021.104570.
22. Matarese, A.; Gambardella, J.; Sardu, C.; Santulli, G. MiR-98 Regulates TMPRSS2 Expression in Human Endothelial Cells: Key Implications for COVID-19. *Biomedicines* **2020**, *8*, 462, doi:10.3390/biomedicines8110462.
23. Barreda-Manso, M.A.; Nieto-Díaz, M.; Soto, A.; Muñoz-Galdeano, T.; Reigada, D.; Maza, R.M. In Silico and In Vitro Analyses Validate Human MicroRNAs Targeting the SARS-CoV-2 3′-UTR. *Int. J. Mol. Sci.* **2021**, *22*, 6094, doi:10.3390/ijms22116094.

24. Centa, A.; Fonseca, A.S.; Ferreira, S.G. da S.; Azevedo, M.L.V.; Vaz de Paula, C.B.; Nagashima, S.; Machado-Souza, C.; Miggiolaro, A.F.R.D.S.; Baena, C.P.; de Noronha, L.; et al. Deregulated MiRNA Expression Is Associated with Endothelial Dysfunction in Post-Mortem Lung Biopsies of COVID-19 Patients. *Am. J. Physiol. Lung Cell. Mol. Physiol.* **2020**, doi:10.1152/ajplung.00457.2020.
25. Gasparello, J.; Finotti, A.; Gambari, R. Tackling the COVID-19 "Cytokine Storm" with MicroRNA Mimics Directly Targeting the 3'UTR of pro-Inflammatory MRNAs. *Med. Hypotheses* **2021**, *146*, 110415, doi:10.1016/j.mehy.2020.110415.
26. Balmeh, N.; Mahmoudi, S.; Mohammadi, N.; Karabedianhajiabadi, A. Predicted Therapeutic Targets for COVID-19 Disease by Inhibiting SARS-CoV-2 and Its Related Receptors. *Inform. Med. Unlocked* **2020**, *20*, 100407, doi:10.1016/j.imu.2020.100407.
27. Lu, D.; Chatterjee, S.; Xiao, K.; Riedel, I.; Wang, Y.; Foo, R.; Bär, C.; Thum, T. MicroRNAs Targeting the SARS-CoV-2 Entry Receptor ACE2 in Cardiomyocytes. *J. Mol. Cell. Cardiol.* **2020**, *148*, 46–49, doi:10.1016/j.yjmcc.2020.08.017.
28. Fernández-Pato, A.; Virseda-Berdices, A.; Resino, S.; Ryan, P.; Martínez-González, O.; Pérez-García, F.; Martin-Vicente, M.; Valle-Millares, D.; Brochado-Kith, O.; Blancas, R.; et al. Plasma MiRNA Profile at COVID-19 Onset Predicts Severity Status and Mortality. *Emerg. Microbes Infect.* **2022**, *11*, 676–688, doi:10.1080/22221751.2022.2038021.
29. Eichmeier, A.; Kiss, T.; Kocanova, M.; Hakalova, E.; Spetik, M.; Cechova, J.; Tichy, B. Conserved MicroRNAs in Human Nasopharynx Tissue Samples from Swabs Are Differentially Expressed in Response to SARS-CoV-2. *Genes* **2022**, *13*, 348, doi:10.3390/genes13020348.
30. Elemam, N.M.; Hasswan, H.; Aljaibeji, H.; Sharif-Askari, N.S.; Halwani, R.; Taneera, J.; Sulaiman, N. Profiling Levels of Serum MicroRNAs and Soluble ACE2 in COVID-19 Patients. *Life* **2022**, *12*, 575, doi:10.3390/life12040575.
31. Zhang, S.; Amahong, K.; Zhang, C.; Li, F.; Gao, J.; Qiu, Y.; Zhu, F. RNA–RNA Interactions between SARS-CoV-2 and Host Benefit Viral Development and Evolution during COVID-19 Infection. *Brief. Bioinform.* **2022**, *23*, bbab397, doi:10.1093/bib/bbab397.
32. Bartoszewski, R.; Dabrowski, M.; Jakiela, B.; Matalon, S.; Harrod, K.S.; Sanak, M.; Collawn, J.F. SARS-CoV-2 May Regulate Cellular Responses through Depletion of Specific Host MiRNAs. *Am. J. Physiol. - Lung Cell. Mol. Physiol.* **2020**, *319*, L444–L455, doi:10.1152/ajplung.00252.2020.
